# Dual Role of Silver Moieties Coupled with Ordered Mesoporous Cobalt Oxide towards Electrocatalytic Oxygen Evolution Reaction

**DOI:** 10.1002/anie.202003801

**Published:** 2020-07-21

**Authors:** Mingquan Yu, Gun‐hee Moon, Rebeca G. Castillo, Serena DeBeer, Claudia Weidenthaler, Harun Tüysüz

**Affiliations:** ^1^ Max-Planck-Institut für Kohlenforschung Kaiser-Wilhelm-Platz 1 45470 Mülheim an der Ruhr Germany; ^2^ Max Planck Institute for Chemical Energy Conversion Stiftstrasse 34–36 45470 Mülheim an der Ruhr Germany

**Keywords:** activation, Ag_2_O nanoclusters, iron incorporation, ordered mesoporous Co_3_O_4_, oxygen evolution reaction

## Abstract

Herein, we show that the performance of mesostructured cobalt oxide electrocatalyst for oxygen evolution reaction (OER) can be significantly enhanced by coupling of silver species. Various analysis techniques including pair distribution function and Rietveld refinement, X‐ray absorption spectroscopy at synchrotron as well as advanced electron microscopy revealed that silver exists as metallic Ag particles and well‐dispersed Ag_2_O nanoclusters within the mesostructure. The benefits of this synergy are twofold for OER: highly conductive metallic Ag improves the charge transfer ability of the electrocatalysts while ultra‐small Ag_2_O clusters provide the centers that can uptake Fe impurities from KOH electrolyte and boost the catalytic efficiency of Co–Ag oxides. The current density of mesostructured Co_3_O_4_ at 1.7 V_RHE_ is increased from 102 to 211 mA cm^−2^ with incorporation of silver spices. This work presents the dual role of silver moieties and demonstrates a simple method to increase the OER activity of Co_3_O_4_.

## Graphical abstract

**One stone, two birds**: The twofold advantages of silver coupling with ordered mesoporous Co_3_O_4_ for OER are demonstrated. Metallic silver enhances the conductivity of the structure while Ag_2_O nanoclusters provide centers for Fe uptake from the KOH electrolyte and boost significantly the efficiency of the electrocatalyst.

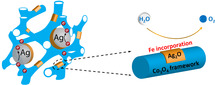

## Introduction

As part of promising future hydrogen economy, water electrolysis has been intensively studied as an economically viable and environment‐friendly technology for hydrogen production.^1^, ^2^ Electrochemical water splitting has distinct advantages, in particular a high conversion efficiency of ≈60 % which is comparable to that of conventional steam reforming technologies, as well as the potential to mitigate intermittency issues associated with utilization of electricity from the renewable energy sources (such as solar and wind power).^3^, ^4^ Although hydrogen is produced by the reductive pathway, it is essential to minimize the overpotential of the oxidative half reaction since the oxygen evolution reaction (OER) proceeds through the sluggish multiple proton‐coupled electron transfer and rigid oxygen‐oxygen bonding. Up to now, the best‐known OER catalysts are based on iridium and ruthenium oxide, but the scarcity, high cost, and poor durability hinder their practical applications.^5^ Therefore, research interest has been focused on developing alternative electrocatalysts either consisting of less expensive elements (e.g., transition metals, modified carbon materials, etc.),^6^, ^7^, ^8^ or containing a small amount of noble metals as dopants or single atom catalysts.^9^, ^10^ Among transition‐metals‐based OER catalysts such as oxides,^5^, ^11^ (oxy)hydroxides,^12^ sulfides,^13^ phosphates,^14^ and nitrides,^15^ spinel cobalt oxides (Co_3_O_4_) with a relatively good OER performance are one of the promising candidates owing to its earth abundance, sustainability against corrosion, ease of control of morphologies and outstanding redox capability.^16^ Nevertheless, there is still significant room to improve the OER activity of Co_3_O_4_ electrocatalyst.

There are three main approaches to enhance activities of electrocatalysts, namely (i) increasing the number of active sites, (ii) improving the intrinsic property of each active center by tuning their electronic structures, and (iii) enhancing the electrical conductivity by hybridization with carbon based materials.^17^, ^18^ Co_3_O_4_ structure can be straightforwardly tuned to boost the surface catalytic performance by generating more accessible sites.^19^, ^20^, ^21^ For instance, we have employed a nanocasting route to construct mesoporous metal oxides with high surface area and excellent structural stability against collapsing pores or channels due to oxygen bubbling during the OER process.^16^, ^22^ Incorporation of hetero‐atoms at the dopant level can tune the electronic state and intrinsic activity of the electrocatalyst.^23^ Numerous studies have shown that the OER activity of spinel Co_3_O_4_ can be increased by incorporating transition metals (e.g., Ni, Fe, Cu, Cr, etc.) into the Co_3_O_4_ structure,^20^, ^24^ whereas doping Mn reduces the catalytic activity.^16^, ^25^ Carbon materials have been widely utilized as an additive to increase the poor conductivity of transition metal oxides, for example, Co_3_O_4_ suffers from intrinsic semiconducting behavior in electrochemical reactions,^26^ thus it is essential to lower the resistance at catalyst‐electrode interfaces.^27^ The drawbacks of carbon is its unavoidable oxidation during the OER process, which leads to either the decrease of conductivity or the deactivation of active sites on electrocatalysts.^28^

Silver particles are frequently used as main components of conductive paste to increase the conductivities of metal oxides for diverse applications.^29^ Silver has the highest electrical conductivity (6.30×10^7^ S m^−1^ at 20 °C) among all metals,^30^ and it should be a good candidate to increase the intrinsic activity of cobalt oxide for OER.^31^, ^32^ With a view to sustainability and cost, silver has a robust electrochemical stability in alkaline conditions and a relatively reasonable price compared to rare‐earth elements, including noble metals. Therefore, engineering Co_3_O_4_ structure via coupling with conductive Ag particles could be employed as an effective strategy to improve its electrocatalytic performance.^32^, ^33^, ^34^, ^35^, ^36^ Obviously, the formation of metallic Ag is beneficial for the electrical conductivity of Co_3_O_4_.^35^, ^36^, ^37^, ^38^ Moreover, recently Zeng et al. observed that less than 1 % of Ag^+^ cations addition to CoSe_2_ could facilitate the charge transfer process that serves as the key step in electrocatalysis.^31^

Herein, we used the ordered mesoporous (OM) structure as a model system to explore the synergistic effect between cobalt and silver in spinel cobalt oxides with the aim of developing high‐performance OER electrocatalysts. By following the hard templating approach, silver could be incorporated into mesostructured Co_3_O_4_ in two phases, namely metallic nanoparticles and ultra‐small Ag_2_O clusters. The synergy of these two silver species resulted in enhanced OER performance by reaching a much lower overpotential (371 mV) at 10 mA cm^−2^ than that of the pristine Co_3_O_4_ (392 mV). Moreover, the overpotential further decreased 344 mV at 10 mA cm^−2^ after chronopotentiometry experiment, which is similar to what occurs when amorphous cobalt oxide is activated by iron impurities.^24^ Various physical characterization and electrochemical data support that the Ag_2_O nanoclusters provided the sites to uptake trace iron impurities from KOH electrolyte, which stimulated the kinetics in terms of lowering the activation energy barrier for water oxidation. To the best of our knowledge the present results represent the first time that the dual‐functional role of silver moieties coupled with OM Co_3_O_4_ has been elucidated.

## Results and Discussion

**Figure 1:**
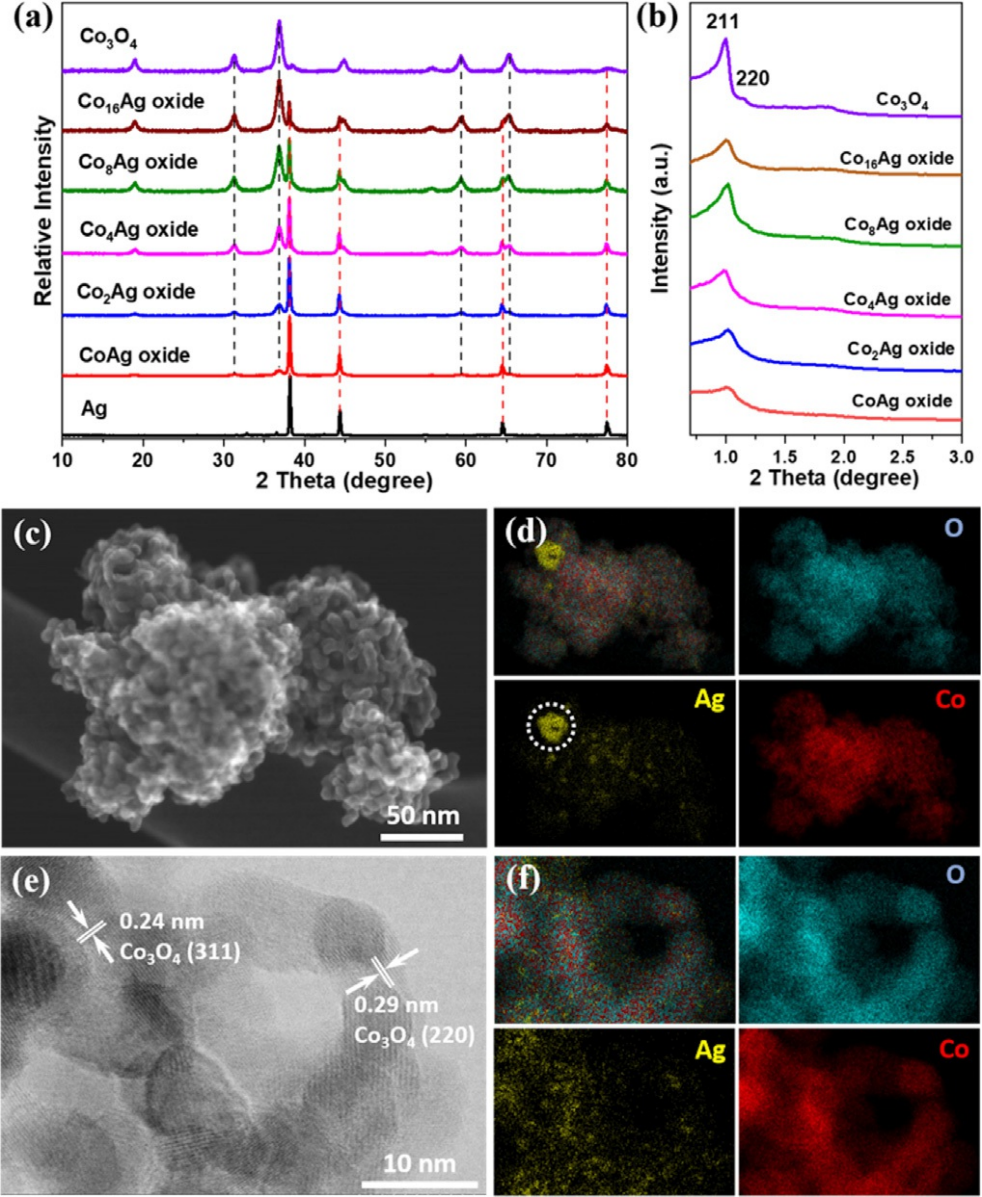
a) Wide‐angle XRD patterns, b) small‐angle XRD patterns of nanocast Ag‐Co oxides. The characteristic reflections of Co_3_O_4_ spinel (PDF: 042‐1467) and metallic Ag (PDF: 004‐0783) were marked by black and red dash lines in (a), respectively. HR‐SEM image (c), HR‐TEM image (e), and the corresponding elemental mapping images (d) and (f) of nanocast Co_8_Ag oxide, respectively. The marked circle in Ag mapping in (d) represents the large metallic Ag particle embedded in the OM‐Co_3_O_4_ matrix.

Following a well‐developed nanocasting protocol,^39^ a series of Ag‐Co oxides with Co/Ag atomic ratios of 16, 8, 4, 2 and 1 were prepared by using cubic OM KIT‐6 silica as a hard template. Synthetic details are given in experimental section of supporting information. Powder X‐ray diffraction (XRD) patterns shown in Figure 1 a confirm the formation of Co_3_O_4_ spinel and metallic Ag for all mixed samples. Notably, compared to Co_3_O_4_ spinel, metallic Ag exhibited much sharper diffraction patterns, suggesting its high crystallinity with a large crystallite size. During the thermal treatment, the starting precursors of cobalt and silver nitrates are converted to their oxide and metallic counterparts. Although the passivated silver and silver oxide are stable at the ambient condition, at high temperature as 550 °C, Ag_2_O further decomposes to Ag and oxygen (2 AgNO_3_=Ag_2_O + 2 NO_2_ + 0.5 O_2_; Ag_2_O=2 Ag + 0.5 O_2_).^40^ Sintering and agglomeration of the small silver clusters cause the formation of metallic silver particles within 3D Co_3_O_4_ mesostructure. This can be also seen from the transmission electron microscopy (TEM) images of the nanocast oxides. As shown in Figure S1, an OM Co_3_O_4_ structure with an average crystallite size of around 7 nm (which is corresponding to the pore size of silica template) is obtained after the removal of silica template, illustrating a successful replication process. By simply tuning the composition of the starting precursor during the impregnation step, a series of cobalt–silver oxides with designed Co/Ag ratio were successfully synthesized as well. The actual ratio of Co/Ag was determined by energy‐dispersive X‐ray spectroscopy (EDX, Figure S2), and the results are listed in Table S1. A lower amount of Ag was always observed in the nanocast Ag‐Co oxides compared to the designed ratios. Especially for the oxide with high Ag loading, for example, the oxide with a Co/Ag ratio of 1/1, ≈36 at. % of Ag was lost during the preparation steps. This can be ascribed to: (1) limited solubility of AgNO_3_ in ethanol (3.1 g/ 100 g, 18 °C),^41^ (2) slight dissolution of Ag in hot base solution, and (3) diffusion of excess AgNO_3_ out of the silica template, followed by decomposition to form Ag on crucible during the calcination.

The well‐ordered mesoporous structure of KIT‐6 silica template was replicated to some extent on the Ag–Co oxides, although the structure looks denser and coupled as seen in Figure S3. This is due to the formation of large Ag particles, which were well integrated with the OM Co_3_O_4_ matrix during nanocasting. As shown in Figure S4a, a large particle of ≈100 nm was embedded within the oxide sample. High‐resolution TEM image shows the lattice fringes on the large crystal with a spacing of 0.20 nm, corresponding to the (200) plane of Ag metal (Figure S4b and S4c). Good contact existed between the large Ag crystallites and the OM matrix. The nanoparticles of the OM matrix are Co_3_O_4_ nanoparticles, as suggested by the characteristic lattice fringes observed at different positions (Figure S4c–e). As a result, the cubic symmetry of the ordered mesoporous structure is distorted with the addition of Ag, as displayed by the lower peak intensities in Figure 1 b. The addition of Ag during nanocasting does not only change the morphology but also significantly influence the textural parameters. The textural parameters summarized in Table S1 show that, the values of both Brunauer‐Emmett‐Teller (BET) surface area and pore volume exhibit a decreasing trend with an increasing amount of Ag (Figure S5). Obviously, this is due to the formation of Ag particles with larger crystal size as well as higher density, which is in agreement with the XRD and TEM results.

A series of systematic characterizations were performed on a selected oxide (Co/Ag 8/1) to investigate the structural information of Ag as well as its distribution in the OM Co_3_O_4_ matrix. The high‐resolution scanning electron microscopy (HR‐SEM) image and the EDX mapping images show the uniform distribution of O and Co (Figure 1 c and d), while some Ag is concentrated on an aggregate, supporting the formation of metallic Ag particles as already observed by XRD. In addition, small amounts of Ag were also homogeneously dispersed over the entire sample, which might give a hint for the formation of a secondary silver specie. To confirm this, we took a closer observation on the architecture with high resolution images, and an area comprising of small nanoparticles (≈7 nm) was checked (Figure S6). The lattice fringes with a spacing of 0.24 and 0.29 nm correspond to the (311) and (220) planes of Co_3_O_4_ spinel, respectively (Figure 1 e). Over the individual Co_3_O_4_ particles, homogeneously distributed Ag species are still observed in the TEM‐EDX images (Figure 1 f). Furthermore, spot EDX analysis was carried out on representative locations to check the local elemental composition. As shown in Figure S7, concentrated Ag (over 60 at. %) was found in the area containing large Ag particle, whereas a small amount of Ag (1.3–2.5 at. %) was detected where well‐defined Co_3_O_4_ mesostructure is present. These results demonstrate that bulky metallic Ag aggregates and secondary Ag species with a nanometer scale coexist in/on the mesoporous Co_3_O_4_ structure.

**Figure 2:**
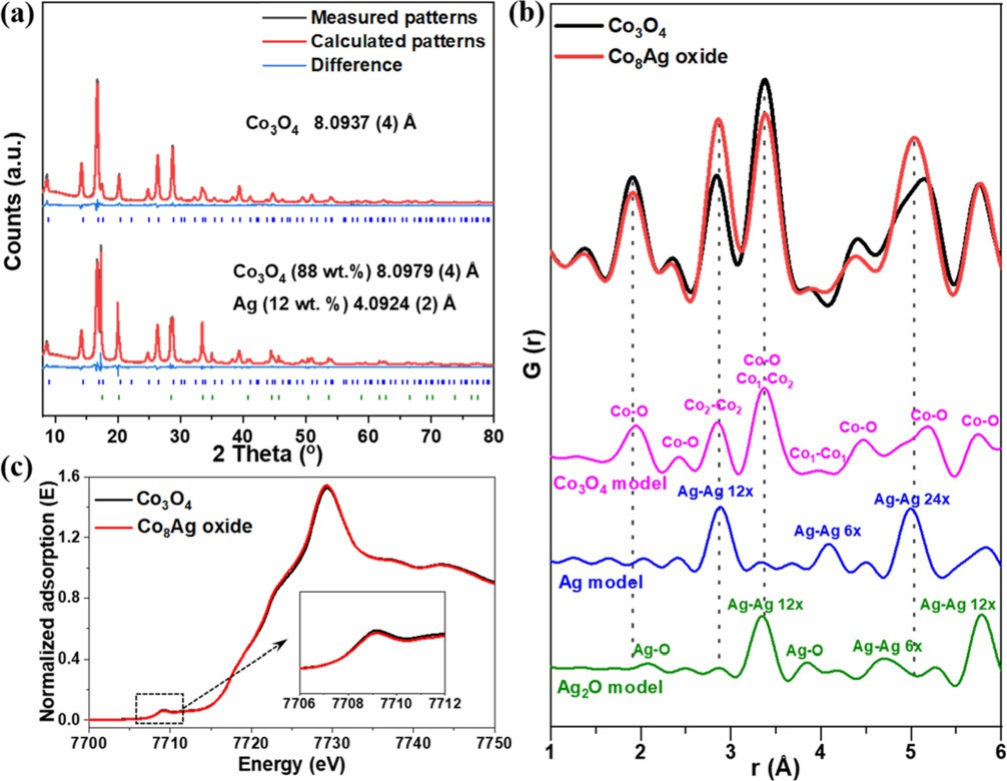
Rietveld refinement analysis of XRD data (a), PDF analysis (b), and Co K‐edge XANES spectra (c) of nanocast Co_3_O_4_ and Co_8_Ag oxide. The model crystal structures of spinel Co_3_O_4_, cubic Ag and Ag_2_O are given in Figure S8. In model spinel structure, Co_1_ and Co_2_ represent tetrahedral and octahedral cobalt sites, respectively.

Although it is well‐known that Co_3_O_4_ spinel can host other transition metals via the substitution on its tetrahedral and octahedral sites,^16^, ^20^, ^25^ it is unlikely to incorporate Ag cation into a compact spinel crystal based on Goldschmidt's rule, since the radius of Ag cations (≈1 Å) is much larger in comparison to Co cations (below 0.75 Å).^42^ We carried out several bulk analysis techniques to explore the silver species and to exclude the possibility of Ag incorporation into the structure of Co_3_O_4_. First, Rietveld refinement was conducted on XRD patterns to obtain a deeper insight on the bulk crystal structure (Figure 2 a). As expected, the nanocast Co_3_O_4_ sample is phase pure with the standard spinel structure, while Co_8_Ag oxide consists of a mixture of Co_3_O_4_ (88 wt. %) and metallic Ag (12 wt. %). The unchanged unit cell parameter of Co_3_O_4_ in Co_8_Ag oxide illustrates that no significant expansion of the unit cell is observed for Co_3_O_4_ structure. Second, to better reveal the structural environment, pair distribution function (PDF) analysis was performed and demonstrated well‐resolved peaks of local bonding in Co_3_O_4_ spinel structure, as well as the distinct Ag–Ag bonding occurring in the structure of metallic Ag (Figure 2 b). Notably, the atom spacing of local Co‐O, Co‐Co remained the same, further suggesting the local structural environment in Co_3_O_4_ was not influenced by the presence of Ag. Last, we employed X‐ray absorption spectroscopy (XAS) to deduce the coordination number and valence state of local Co spinel structure by studying both X‐ray absorption near edge structure (XANES) and extended X‐ray absorption fine structure (EXAFS). As shown Figure 2 c, nearly identical Co K‐edge XANES spectra were measured on Co_3_O_4_ and Co_8_Ag oxide, demonstrating that Ag brought a negligible change in the local structure of Co coordination. No significant change on the bonding lengths between Co and neighboring atoms, as shown by EXAFS spectra in Figure S9, illustrates that the coordination environment of Co is maintained upon the addition of Ag. These results clearly support that Ag atoms were not incorporated in the Co_3_O_4_ spinel (i.e., no substitution of Co by Ag) but rather homogeneously distributed on the surface of Co_3_O_4_.

**Figure 3:**
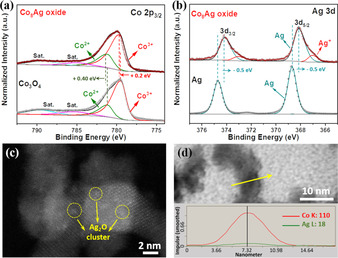
a) High‐resolution XPS spectra of Co 2p of nanocast Co_3_O_4_ and Co_8_Ag oxide. b) High‐resolution XPS spectra of Ag 3d of nanocast Ag and Co_8_Ag oxide. HAADF‐STEM image (c) and line‐scan EDX elemental distribution curves of Co and Ag (d) for Co_8_Ag oxide.

To obtain further insight into the surface structure of Co_3_O_4_ and Co_8_Ag oxide, X‐ray photoelectron spectroscopy (XPS) was carried out. As shown in the high‐resolution Co 2p spectra (Figure 3 a), characteristic peaks of Co^2+^ and Co^3+^ were fitted for the Co 2p_3/2_ peak for Co_3_O_4_ and Co_8_Ag oxide.^21^, ^43^ For a pure reference Ag sample, the Ag 3d region shows a typical spectrum comprising two peaks which can be assigned to Ag 3d_1/2_ and Ag 3d_3/2_ spin‐orbit levels of Ag^0^.^32^, ^44^ While for Co_8_Ag oxide, additional peaks were observed with the binding energy ascribed to Ag^+^ in Ag_2_O compound.^34^ By taking into account that the ultrasmall silver particles or clusters could be easily oxidized, homogeneously dispersed Ag shown in Figure 1 f should belong to the nanoclusters of Ag_2_O. It is worth mentioning that a slight shift was shown on the bonding energy of both metal elements, with a positive shift and negative shift on Co and Ag, respectively, suggesting electron transfer from Ag to Co. Such electron transfer can be explained by the difference in work function between Ag (4.26 eV) and Co in Co_3_O_4_ (4.5 eV),^45^ which forms due to the Coulomb or polaronic interactions between Ag particles and Co_3_O_4_ matrix. As a result, this contact of the Ag/Co_3_O_4_ interface caused surface electron dislocation, as demonstrated by XPS, but not shown in bulk sensitive XAS measurement. Furthermore, high‐angle annular dark field (HAADF) image shows that Ag_2_O is located on the surface of Co_3_O_4_ as ultrasmall nanoclusters (<2 nm). The Ag_2_O nanoclusters can be distinguished from Co_3_O_4_ on the basis of the brightness variations of these nanoclusters in the HAADF‐STEM image (Figure 3 c). As indicated with yellow circles in Figure 3 c, the Ag_2_O clusters are well dispersed on the surface of Co_3_O_4_, in good agreement with the observation from elemental mapping images (Figure 1 d and f). The local structure of Ag_2_O was not observed in the PDF analysis (Figure 2 b), which is due to the low concentration (1.3–2.5 at. % as shown in spot EDX analysis, Figure S7). The existence of Ag_2_O clusters on OM Co_3_O_4_ is further supported by the EDX line‐scanning profile, where small amount of Ag species were examined throughout an individual Co_3_O_4_ nanoparticle (Figure 3 d).

**Figure 4:**
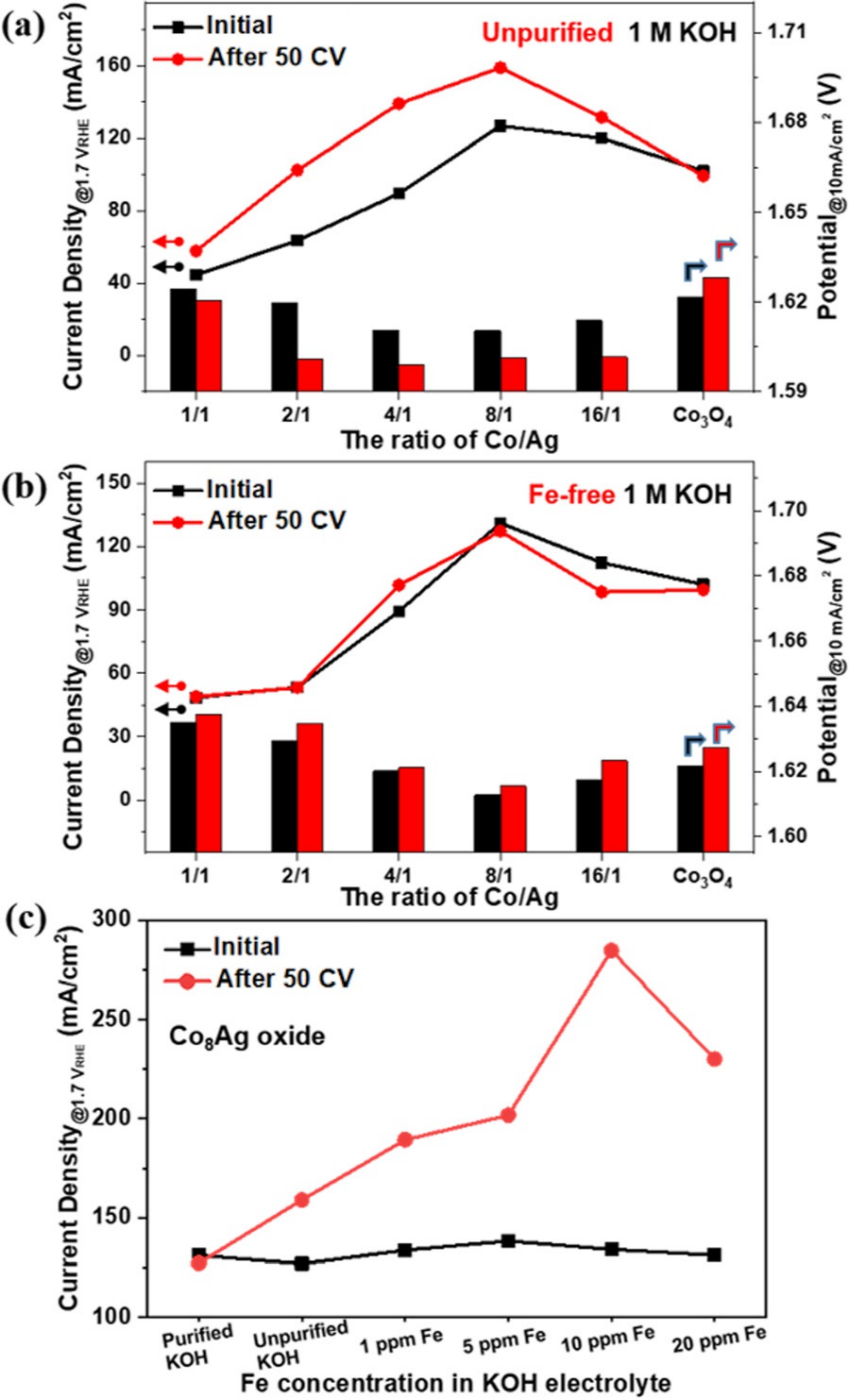
Current density at 1.7 V_RHE_ (lines, left axis) and applied potential at 10 mA cm^−2^ (columns, right axis) for Ag‐Co oxides. The results in (a) and (b) were obtained in 1 m KOH electrolyte with trace Fe impurities and Fe‐free 1 m KOH after purification, respectively. c) Current density at 1.7 V_RHE_ of Co_8_Ag oxide in 1 m KOH electrolyte containing varying Fe concentration. Since KOH pellets containing up to 10 ppm Fe impurity was utilized to prepare 1 m KOH electrolyte, a trace amount of Fe below 0.5 ppm is expected in unpurified KOH electrolyte.

After a detailed structural analysis, the synergy of coupling silver with cobalt is further investigated for OER in 1 m KOH electrolyte. The nanocast Ag–Co oxides were deposited on glassy carbon electrodes and their efficiencies were evaluated following the protocol proposed by Jaramillo and co‐works.^5^ Two important catalytic parameters, namely, current density at 1.7 V_RHE_ and required potential to reach 10 mA cm^−2^, are summarized in Figure 4 a, and show that the catalytic activity has an obvious dependence on the material composition. Superior activity was achieved with the addition of small amount of Ag (up to 20 mol. %), illustrating the beneficial effect of Ag on the OER activity of Co_3_O_4_. Further increasement of silver results in a lower OER activity. This can be ascribed to the lower surface area as well as the significantly decreased cobalt amount in the mixed oxides. With comparing linear sweep voltammetry (LSV) curves collected before and after 50 cycle voltammetry (CV) scans (0.6–1.6 V_RHE_), we can deduce the activation/deactivation behaviors of samples under applied potentials in KOH electrolyte. As seen in Figure S10 and S11, pristine Co_3_O_4_ shows stable OER activity, whereas a higher catalytic current is achieved on Ag‐containing oxides after 50 CV scans, suggesting the activation of samples. This can be also seen in Figure 4 a, where higher current densities as well as lower overpotentials were achieved in terms of the electrochemical activation of Ag‐Co oxides by the CV scans. Among these oxides, Co_8_Ag oxide shows the highest OER activity after activation with a current density (159 mA cm^−2^) and an overpotential (371 mV), which are superior to pristine Co_3_O_4_ (99 mA cm^−2^ and 398 mV).

In order to understand the electrochemical activation of Ag‐Co oxides, we conducted the same experiment in Fe‐free KOH electrolyte, since trace Fe impurities present in the electrolyte are known as a booster to enhance the OER activity in the case of nickel/cobalt based oxide catalysts.^24^, ^43^, ^46^, ^47^, ^48^ As expected, irrespective to the presence of Ag, the activation was not observed at all in Fe‐free electrolyte (Figure 4 b, S12 and S13). Instead, a slight deactivation was observed, which could be attributed to the detachment of the catalyst film or the increase of the resistance by the oxidation of metallic silver.^49^ This comparison measurement illustrates that Fe impurities present in the electrolyte are essential for the activation profile of the Ag–Co oxides. On the other hand, the sample with a Co/Ag (8:1) ratio still showed the highest activity compared with other Ag–Co oxides. A similar activity trend was observed from the electrochemical measurements in both Fe‐containing and Fe‐free KOH electrolytes, with Co_8_Ag oxide being the most active OER catalyst. Clearly, the addition of Ag is beneficial to the OER activity of spinel Co_3_O_4_, as shown in previous studies where Ag was employed as a conductive additive.^31^, ^32^ Since the Ag–Co oxides have the same elemental composition (i.e., consisting of mostly Ag, Co, and O but with a different ratio), it is reasonable to postulate that either the nature of active species or the number of active sites should be responsible for the high OER activity of Co_8_Ag oxide. Accordingly, relevant experiments were conducted to verify this postulation.

To elucidate the origin of superior OER activity on Co_8_Ag oxide, the following experiments were carried out to compare the Ag‐Co oxides with a different Co/Ag ratio. First, we normalized the LSV curves based on the specific surface areas which were determined by N_2_ physisorption measurement. As shown in Figure S14, the Co_8_Ag oxide brought a significant higher current density at 1.7 V_RHE_, illustrating that it is intrinsically more efficient at uptaking electrons from OH^−^ to generate oxygen. Second, we investigated the correlation between the catalytic performance and the charge transfer ability that is a crucial factor in electrocatalysis. For this, the electrochemical impedance spectroscopy (EIS) measurement was conducted to unveil the reaction kinetics on these oxides. Figure S15 depicts the Nyquist plots measured at 1.6 V_RHE_, from which we can derive the corresponding resistances according to Randle model (Figure S16). As expected, introducing Ag significantly decreased the charge transfer resistance (based on the diameter of the semicircle in the low‐frequency region). Among the Ag‐containing oxides, similar values of charge transfer resistance (16–19 ohm) are shown except for CoAg oxide which possesses relatively higher value (28 ohm). The high resistance of CoAg oxide could be resulted from the numerous points of contact interconnected between particle domains in a collapsed ordered structure.^50^ Besides the contribution from metallic Ag particles, it has been reported that trace Fe impurities can be electrochemically incorporated into cobalt based oxides during the electrochemical measurement, resulting in a significant increasement on the charge transfer kinetics of the OER.^43^, ^51^, ^52^ To rule out the effect of the electrochemical Fe uptake on OER, we also performed the EIS measurement in Fe‐purified KOH electrolyte. As seen in Figure S15b, higher charge transfer resistances were confirmed in all samples, supporting that Fe is an important element to reduce the activation energy barrier to split water in Ag‐Co oxide systems. And now, it is evident to see the effect of Ag on the charge transfer ability of Ag‐Co oxides, where the Co_8_Ag oxide possesses the lowest charge transfer resistance, in other words the highest intrinsic property. This contributes to the superior OER activity on Co_8_Ag oxide over other Co–Ag oxides in the absence of Fe uptake. Furthermore, we measured the double‐layer capacitance (C_dl_) to estimate the electrochemical surface area (ECSA) of nanocast Co–Ag oxides upon activation. As shown in Figure S17 and S18, the electrochemical surface area (ECSA) of all samples were similar except Co_2_Ag and CoAg oxides with much lower BET surface areas (Figure S5). The slightly higher value of C_dl_ for Co_4_Ag, Co_8_Ag, and Co_16_Ag oxides relative to Co_3_O_4_ could be due to the compensation of their lower BET surface area by the electron storing behavior of silver.

In addition to the improved OER kinetics, it has been recently demonstrated that Fe uptake leads to the formation of oxidized Fe species, which plays a crucial role in lowering the activation energy barrier for the OER.^53^, ^54^ At the same time, the electron transfer can be stimulated via the Fe uptake or the electrochemical Fe deposition from the unpurified KOH electrolyte.^43^, ^53^, ^55^ According to our previous work, the oxygen evolution kinetics on NiCo oxides were markedly enhanced through the uptake of Fe impurities.^43^ In the present study, a similar effect was observed in which Ag–Co oxides were activated upon the electrochemical measurement in Fe‐contaminated KOH electrolyte. As a result, the induced Fe favors the transfer of electrons more efficiently from OH^−^ reactant to electrode, thus expediting the OER reaction. In addition, it was found that the catalytic behavior of Co_8_Ag oxide is affected by the concentration of Fe impurity in KOH electrolyte. As shown in Figure S19, Co_8_Ag electrocatalyst was activated in all Fe‐containing KOH electrolytes. Depending on the Fe content, varying OER activities were shown after Fe‐activation. Figure 4 c shows that maximum activation was achieved in 1 m KOH electrolyte that contains 10 ppm Fe impurity, further supports that Fe uptake induced activity enhancement on Co‐Ag oxides.

In Co‐based electrocatalytic systems, it has been proposed that the formation of active species could be ascribed by (i) Fe‐modified Co sites,^56^, ^57^ (ii) Fe species with a high‐valent oxidation state,^51^, ^54^ and (iii) coordinated Co–Fe moiety.^52^ Although it is still controversial, there is no doubt that the efficient uptake of Fe impurities is essential to activate Co‐based electrocatalysts for OER.^24^, ^43^, ^51^, ^52^ Our recent study also confirmed that the capability to uptake Fe depends on the structure of cobalt‐based electrocatalysts, where amorphous Co(OH)_2_ and CoOOH were able to uptake Fe impurities from the electrolyte and thus got activated.^24^ However, the trend that the Fe‐induced activation was not proceeded in crystalline Co_3_O_4_ electrocatalysts indicates that the spinel structure is not favorable to uptake Fe impurities from KOH electrolyte.^24^, ^43^ This is also seen in the present work, where slight deactivation was observed on pristine Co_3_O_4_ in both unpurified and Fe‐free KOH electrolyte (Figure 4). Hence, the silver sites appear to impart a means to uptake Fe impurities for the activation, which is an unknown phenomenon that to our knowledge has not been previously reported.

**Figure 5:**
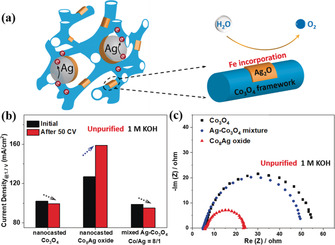
a) Schematic illustration of structure of Co_8_Ag oxide, demonstrating the role of Ag metallic particles and Ag_2_O clusters, b) A comparison of current density at 1.7 V_RHE_ collected before and after 50 CV, and c) Nyquist plots of nanocast Co_3_O_4_, Co_8_Ag oxide, and mixed Ag‐Co_3_O_4_ with a Co/Ag ratio of 8/1. The measurements were performed in unpurified 1 m KOH electrolyte where Fe impurities were present.

On the basis of the detailed characterizations, it is confirmed that two Ag species are formed with the introduction of Ag, which are: (i) metallic Ag particles coupled with OM Co_3_O_4_ matrix that can act as a good electron conductor, (ii) ultrasmall Ag_2_O clusters homogeneously dispersed on the surface of Co_3_O_4_ particles that provide the sites for uptaking Fe from KOH electrolyte as schematically illustrated in Figure 5 a. To understand the role of both Ag metal and Ag_2_O cluster on the OER activity of Co_8_Ag oxide, we prepared the sample by physically mixing with pure Ag and the OM Co_3_O_4_ (Co:Ag 8:1). As seen in Figure 5 b, among these three samples, the highest OER activity and Fe‐induced activation were revealed on nanocast Co_8_Ag oxide. Constructing a hetero‐interface with Ag has been well reported as an effective strategy to increase the electron transfer ability on transition metal based electrocatalysts.^31^, ^38^, ^58^ The resistance of the sample series was further measured through a simple home‐made cell that provides a relative comparison of the resistance of materials. The calculated resistance from the slope of *I*–*V* curves (under bias sweeping from 9 to 1 V) is displayed in Figure S20. The resistance of Co_3_O_4_ is 12.37 MΩ and the value is curtailed and then risen with increasing the atomic ratio of silver to cobalt, the lowest resistance is observed in Co_8_Ag oxide (0.27 MΩ). This trend matches well with the OER activity results (Figure 4 b). As reference materials, the resistances of commercially‐available Ag nanoparticles and Ag_2_O powder were also measured, as it is expected, the metallic silver completely loses its conductivity when oxidized to silver oxide. The result supports that a certain amount of metallic silver is helpful to improve the electron transfer occurring throughout OM Co_3_O_4_ but an excess amount of metallic silver covered by a thin oxide layer hinders the electron transfer due to the insulating behavior of Ag_2_O. After 50 CV, the physical mixture of nanocast Ag and Co_3_O_4_ shows slight deactivation instead of Fe‐induced activation. This indicates that metallic Ag species are not capable to uptake Fe impurities from KOH electrolyte, and it is necessary to add Ag_2_O species for the activation process. A trace amount of Fe impurities (0.18 ppm) was found to be presented in normal 1 m KOH solution, likely in the form of [Fe(OH)_4_]^−^.^52^, ^59^ In principle, positively charged material is capable of adsorbing [Fe(OH)_4_]^−^ species in KOH electrolyte. Although, both Co_3_O_4_ and Ag_2_O are typical *p*‐type semiconductor,^60^, ^61^ the rigid structure of highly crystalline Co_3_O_4_ is not able to accommodate [Fe(OH)_4_]^−^.^24^ Therefore, Ag_2_O nanoclusters are preferred sites for [Fe(OH)_4_]^−^ adsorption and the corresponding iron uptake under the mild condition.^24^ As a result, iron species were involved in electrochemical water oxidation catalysis and induced activation of Ag–Co oxides.

**Figure 6:**
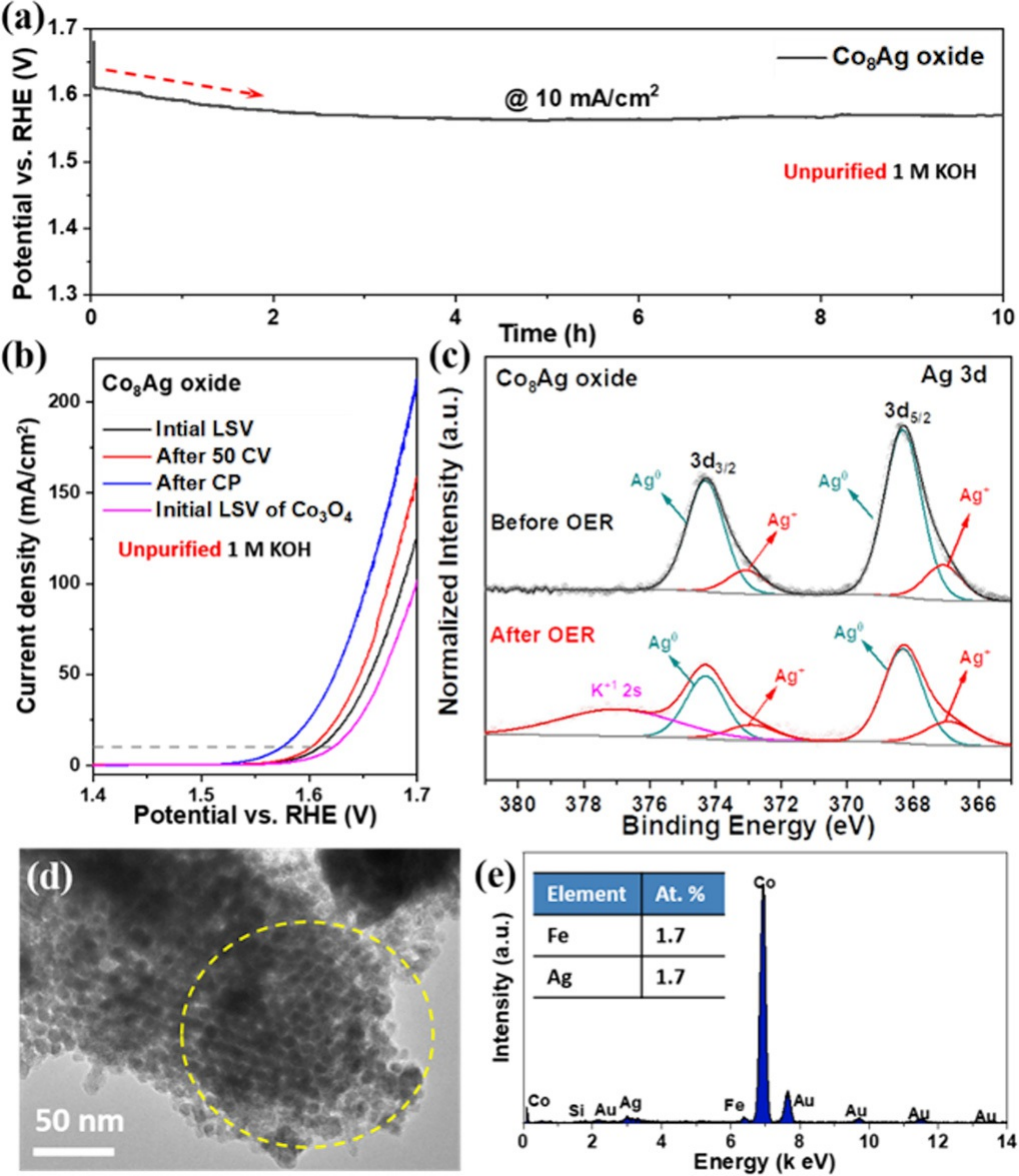
a) Chronopotentiometric (CP) curve at a current density of 10 mA cm^−2^. b) Initial LSV curves, after 50 CV and after CP measurement of Co_8_Ag oxide. XPS spectra of Ag 3d on Co_8_Ag oxide/carbon fiber before and after CP test. d) TEM image of Co_8_Ag oxide after CP measurement. e) local EDX analysis according to the yellow marked circle in (d).

To evaluate the stability performance of nanocast Co_8_Ag oxide, we continuously applied the potential fixed at 10 mA cm^−2^ using a chronopotentiometry (CP) in unpurified 1 m KOH for 10 h (Figure 6 a). The activation stage was observed in the first 2 h with a significant potential decrease, as indicated by the red arrow in Figure 6 a. Subsequently, the potential was stabilized and remained up to 10 h. Increasing Fe content in the electrolyte resulted in an acceleration of activation kinetics. The potential to reach 10 mA cm^−2^ decreased to a steady state around 1.56 V_RHE_ within 10 min when the electrolyte contained 10 ppm Fe (Figure S21). The electrocatalytic performance of Co_8_Ag oxide was checked by LSV scan after CP measurement (Figure 6 b). A further enhancement on the OER activity was achieved with delivering a current density of 10 mA cm^−2^ at an overpotential of 344 mV, which is much lower than those of its initial form (380 mV) and pristine Co_3_O_4_ (392 mV). A lower Tafel slope was calculated on the activated Co_8_Ag oxide (48 mV/dec) compared to that of pristine Co_3_O_4_ (56 mV/dec, Figure S22). This suggests the formation of new surface active species that are kinetically more favorable for OER.^62^ Since Corrigan reported the activation of Ni electrocatalysts caused by iron impurities present in the electrolyte,^46^ numerous operando measurements in combination with DFT calculations have been actively carried out, but the role of iron species is still unclear. One of possible reasons is the formation of active Fe^4+^ species which can serve as dynamically active sites at the catalyst/electrolyte interface with balanced binding energies of oxygen intermediates.^54^, ^63^, ^64^ Another possible reason is the presence of Fe^3+^ stimulating the oxidation of M^2+^ (M: Co and Ni) to M^3+/4+^ instead of its further oxidation to Fe^4+^.^56^, ^57^, ^65^ As seen in Figure S23, the Ag_2_O itself showed a very poor OER activity even in the presence of an excess amount of iron impurity. Therefore, it does make sense to postulate either (i) the interaction between Ag_2_O adsorbed by iron impurities and Co_3_O_4_ or (ii) the formation of new active species consisting of cobalt‐silver‐iron synergistically facilitated the OER kinetics. Resultantly, much higher current density at 1.7 V_RHE_ was achieved on the activated Co_8_Ag oxide (211 mA cm^−2^), more than twice of that of Co_3_O_4_ (102 mA cm^−2^). Even compared with the recent‐reported benchmarked cobalt electrocatalysts, Co_8_Ag oxide displayed competitive catalytic performance, as listed in Table S2.

In addition, post characterizations were conducted to examine the structural durability of the Co_8_Ag electrocatalyst. First, we studied the change in the surface oxidation state of Ag by XPS. For that, the electrode was fabricated by depositing Co_8_Ag oxide on the carbon fiber paper. The survey XPS spectra suggest a similar chemical composition on the electrode before and after electrochemical activation (Figure S24). Both Ag metal and Ag^+^ cations are detected on the surface of the catalyst after OER measurement in KOH electrolyte. However, the peak intensity ratio of Ag^+^/Ag increased significantly, indicating that during the electrochemical measurement metallic silver was converted to silver oxide. It should be noted that the peak at binding energy of 377.0 eV can be ascribed to the remaining K^+^ originating from the electrochemical measurement.^66^ Furthermore, as shown in the TEM image of the Co_8_Ag oxide (Figure 6 d), the well‐ordered mesoporous structure was maintained after CP measurement. On the OM Co_3_O_4_ matrix, 1.7 at. % of Ag was detected by local EDX analysis (Figure 6 e), which could be ascribed to the well‐dispersed Ag_2_O clusters. Nevertheless, the dissolution of Co and Ag was observed on Co_8_Ag electrocatalyst after a long‐term chronopotentiometry (Figure S25 and S26). Electrochemical leaching of transition metals like Ni and Co has been monitored using inductively coupled plasma optical emission spectroscopy in our previous work.^48^ The leaching of Ag can be due to a two‐step oxidation process on metallic Ag particles,^67^ while Ag_2_O clusters remained on Co_3_O_4_ matrix with a similar concentration comparing to as‐prepared sample (1.3–2.5 at. %, Figure S7). Notably, the adsorbed Fe species were also detected on the identical location with a concentration of 1.7 at. %. This observation suggests that equivalent amount of Fe species was adsorbed on Ag_2_O clusters and involved in the OER as catalytically active sites.

## Conclusion

We have fabricated a series of ordered mesoporous silver cobalt oxides and demonstrated that addition of silver has a dual effect on electrocatalytic OER activity of Co_3_O_4_. Following the nanocasting route, two different silver species, namely metallic Ag and ultrasmall Ag_2_O clusters, were formed on/in the mesostructured Co_3_O_4_ matrix. The introduction of metallic Ag increases the conductivity of the electrocatalyst and leads to a faster electron transfer while Ag_2_O species cause the Fe‐induced activation of electrocatalyst. The detailed structural analysis confirmed that metallic Ag particles were grown within the mesostructure of cobalt while Ag_2_O nanoclusters were homogeneously dispersed on the surface of Co_3_O_4_ matrix. Our control experiments and post‐characterization have proven the Fe uptake from the KOH electrolyte through small silver oxide clusters. The adsorbed Fe species improved the OER performance of Co_8_Ag oxide significantly by delivering a current density of 211 mA cm^−2^ at 1.7 V_RHE_ and an overpotential of 344 mV at 10 mA cm^−2^. Increasing Fe content in 1 m KOH electrolyte resulted in a faster activation kinetic where a maximum activation was achieved in the electrolyte containing 10 ppm Fe impurity. This study not only puts forward a direct way to construct active cobalt oxide based electrocatalysts with silver coupling, but also provides experimental evidences on the active role of Fe species originated from KOH electrolyte.

## Conflict of interest

The authors declare no conflict of interest.

## Supporting information

As a service to our authors and readers, this journal provides supporting information supplied by the authors. Such materials are peer reviewed and may be re‐organized for online delivery, but are not copy‐edited or typeset. Technical support issues arising from supporting information (other than missing files) should be addressed to the authors.

Supplementary
