# Supplementary material for: Dual Role of Silver Moieties Coupled with Ordered Mesoporous Cobalt Oxide towards Electrocatalytic Oxygen Evolution Reaction
Source: Angew Chem Int Ed Engl. 2020 Jul 21;59(38):16544–52. doi: 10.1002/anie.202003801 (PMC7540465; doi:10.1002/anie.202003801)
Supplement: Supplementary file 1 — Supplementary [file ANIE-59-16544-s001.pdf]

Angewandte Chemie
Eine Zeitschrift der Gesellschaft Deutscher Chemiker

Supporting Information

Dual Role of Silver Moieties Coupled with Ordered Mesoporous Cobalt Oxide towards Electrocatalytic Oxygen Evolution Reaction

*Mingquan Yu, Gun-hee Moon, Rebeca G. Castillo, Serena DeBeer, Claudia Weidenthaler, and Harun Tüysüz**

anie_202003801_sm_miscellaneous_information.pdf

Experimental Section

Synthesis of KIT-6 hard template. The synthesis of KIT-6 was conducted according to protocol procedures as described before.[1] In detail, 13.5 g of Pluronic 123 (P123, $EO_{20}PO_{70}EO_{20}$, Sigma-Aldrich) was first dissolved in a mix solution of concentrated HCl (37%, 26.1 g) and distilled water, followed by vigorous stirring until P123 was completely dissolved. Then the mixture was placed in an oil bath keeping the temperature at 35 ^oC, and 13.5 g of n-butanol was added into the solution. After 1 h of stirring at 35 ^oC, 29 g of TEOs was quickly added and followed by 24 h stirring. Next, the solution was transferred into an oven and kept at 100 ^oC for another 24 h. Afterwards, the precipitated white powder was filtered and dried overnight at 100 ^oC. The final product was obtained after calcination in air at 550 ^oC for 6 h at a ramping rate of 2 ^oC/min.

Synthesis of ordered mesoporous Co_3O_4 and Ag-Co_3O_4. The ordered mesoporous oxides were prepared via a typical nanocasting using KIT-6 as hard template.[1] Briefly, 0.5 g of KIT-6 was added into 3.6 mL ethanol solution of $Co(NO_3)_2$ $6H_2O$ (Aldrich, 98.0 %) and $AgNO_3$ (Aldrich, 99.0 %) precursors. The ratio of metal precursors was varied as designed with keeping total concentration of 0.8 M. After stirring for 1 h and drying at 80 ^oC overnight, the solid was calcined at 550 ^oC in air for 4 h with a plateau at 250 ^oC for 4 h. The oxides were obtained after leaching out silica template with 2 M NaOH (VWR Chemicals, 85.0 % assay) at 70 ^oC for 12 h.

Purification of KOH electrolyte. Commercial KOH pellets (VWR Chemicals, 85.0 % assay) containing up to 10 ppm Fe impurity were used to prepare normal 1 M KOH solution. To prepare 1 M KOH solution, a trace amount of Fe up to 0.5 ppm is expected in unpurified KOH solution. For Fe-free electrochemical measurements, the KOH electrolyte was purified according to the procedure as described in a recent study from Boettcher's group.[2] In detail, 2 g of high-purity $Ni(NO_3)_2 \cdot 6H_2O$ (Aldrich, 99.999 % trace metals basis) was dissolved in 18.2 MΩ·cm H_2O (4 mL) in a H_2SO_4-cleaned polypropylene (PP) centrifuge tube. 20 mL of 1 M KOH (VWR Chemicals, 85.0 % assay) was added into the tube in order to precipitate high-purity $Ni(OH)_2$. Then, the mixture was shaken for 30 min and the supernatant was decanted after centrifugation. The green solid was then washed three times by adding 18.2 MΩ·cm H_2O (20 mL) and 1 M KOH (2 mL) with redispersing the solid, centrifuging, and decanting the supernatant. The obtained solid was ready for purification of Fe impurities. Briefly, 40 mL fresh-prepared KOH solution (1 M) was added in the tube to re-disperse the high-purity $Ni(OH)_2$ with mechanical shaking for 30 min, followed by 3 h of resting. The mixture was then centrifuged and the KOH supernatant was transferred into a H_2SO_4-cleaned PP bottle for another round of centrifugation. Finally, the purified KOH supernatant was decanted and transferred into a H_2SO_4-cleaned PP bottle for Fe-free electrochemical measurements.

Electrochemical Measurements. Electrochemical measurements were performed in a three-electrode configuration using a rotating disc electrode (Model: AFMSRCE, PINE Research Instrumentation); a hydrogen reference electrode (HydroFlex, Gaskatel) and Pt wire were used as reference electrode and counter electrode, respectively. 220 mL of 1 M KOH solution was filled

in a Teflon cell as electrolyte. Before the electrochemical measurement, argon was purged through the cell for 30 min to remove oxygen. During all measurements, argon was continuously purged to remove generated oxygen and the temperature of the cell was controlled at 25 ^{o}C using a water circulation system. Working electrodes were fabricated by depositing target materials on glass carbon (GC) electrodes (PINE, 5 mm diameter, 0.196 cm^2 area). Before use, all GC electrodes were thoroughly cleaned by polishing with Al_2O_3 suspension (5 and 0.25 μm, Allied High Tech Products, Inc.). For fabricating working electrode, 4.8 mg of powder sample was dispersed in a mixed solution containing 0.75 mL of H_2O, 0.25 mL of 2-propanol (Aldrich, 99.5 %) and 50 μL of Nafion (5% in a mixture of water and alcohol). Afterwards, the mixture solution was immersed in a sonication bath for 30 min to form a homogeneous ink. After that, 5.25 μL of catalyst ink was dropped onto the GC electrode and dried under light irradiation for 10 min. The catalyst loading was calculated to be 0.12 mg/cm^2 in all cases for GC electrodes.

The linear sweep voltammetry (LSV) curves were collected by sweeping the potential from 0.7 V to 1.7 V vs RHE with a scan rate of 10 mV/s. Cyclic voltammetry (CV) measurements were carried out in a potential range between 0.7 and 1.6 vs RHE with a scan rate of 50 mV/s. In all measurements, a rotating disc electrode configuration was kept a rotation speed of 2000 rpm. The IR drop was compensated at 85 % automatically via the potentiostat software (EC-Lab V11.01).

The electrochemical impedance spectroscopy (EIS) was conducted in the same configuration with applying an anodic potential of 1.6 V vs RHE on the glassy carbon electrode. The spectra were collected from 10^5 Hz to 0.1 Hz with an amplitude of 5 mV.

The relative comparison of the resistance for the powder samples was carried out using a homemade cell. Two copper tapes with widths of 7 mm were utilized as an electrode. The gap between two copper tapes that are attached on the teflon holder was 1 mm. 5 mg of the sample was loaded onto the space between the gap of copper tapes and pressurized under 39.2 kPa during the measurement. The current-voltage curves were collected by sweeping the potential from 9 to 1 V using a power supply (2450 SourceMeter, KEITHLEY) for Co_3O_4 and Co_xAg oxide (x = 16, 8, 4, 2, and 1) powder, leading to the calculation of the resistance. As reference materials, the resistance of commercially-available Ag nanoparticles (Aldrich) and Ag_2O powder (Acros Organics) was also measured.

Characterization. Powder X-ray diffraction (XRD) patterns were collected at room temperature on a STOE theta/theta diffractometer in Bragg-Brentano geometry (Cu $K\alpha_{1/2}$ radiation) with a secondary monochromator. N_2-physisorption isotherms were measured using 3Flex Micrometrics at 77 K. Prior to the measurements, the samples were degassed at 150 °C for 10 h. Brunauer-Emmett-Teller (BET) surface areas were determined from the relative pressure range between 0.06 and 0.2. Transmission electron microscopy (TEM) images of samples were measured at 100 kV by an H-7100 electron microscope from Hitachi. High resolution TEM (HR-TEM) and scanning electron microscopy (SEM) images were taken on HF-2000 and Hitachi S-

5500 microscopes, respectively. X-ray photoelectron spectroscopy (XPS) measurements were carried out with a SPECS GmbH spectrometer with a hemispherical analyzer (PHOIBOS 150 1D-DLD). The monochromatized Al Kα X-ray source (E = 1486.6 eV) was operated at 100 W. An analyzer pass energy of 20 eV was applied for the narrow scans. The medium area mode was used as lens mode. The base pressure during the experiment in the analysis chamber was 5 x 10^{-10} mbar. The binding energy scale was corrected for surface charging by use of the C 1s peak of contaminant carbon as reference at 284.5 eV.

Total scattering data for pair distribution function analysis (PDF) were collected on an in-house X-ray powder diffractometer (STOE STADI P) in transmission diffractometer using Mo radiation (0.7093 Å). The instrument is equipped with a primary Ge (111) monochromator ($MoK\alpha_1$) and a position sensitive Mythen1K detector. A generator setting of 50 kV and 40 mA was applied for the generation of X-rays. Data were collected in the range between 5 and 120° 2θ with a step width of 0.015° 2θ. For the measurements, the samples were filled into glass capillaries (Ø 0.5 mm). The program PDFgetX3[3] was used for processing PDFs from the integrated scattering data and PDFgui[4] was used to visualize and simulate PDFs. For correction of background scattering from air and sample container, an empty glass capillary was measured. PDF curves were calculated for Q_{max} of 16 Å^{-1}. Crystal structure data for the simulation of PDFs of Ag, Ag_2O and Co_3O_4 were taken from references.[5-7]

Co K-edge XAS was used to measure nanocast Co_3O_4 and Co_8Ag oxide samples. All samples were prepared as solutions in boron nitride to avoid self-absorption and sealed with 30 µm Kapton tape. Measurements were carried out at beamline 20 BM-B (Static XANES and EXAFS) at Advanced Photon Source (APS). The incident energy was selected by a Si (111) double crystal monochromator. Incident flux was 10^8 photons/seconds using a beam size of 5µm x 5µm and slits size of 3 µm (wide) x 1 µm (high). Samples were kept bellow 20 K in a He displex cryostat. Undamaged data was collected by detuning the incident flux by 15% and stability of the incident beam was monitored by collecting simultaneously Co foil. Incident energy was calibrated by assigning the first inflection point of Co foil to 7709.3 eV. Fluorescence spectra were recorded using a multielement Canberra Ge detector. A step size of 0.3 eV was used in the XANES region (1 sec integration time) and 0.05 Å^{-1} in the EXAFS region to k = 15 Å^{-1} (10-22 seconds integration time). Final spectra were processed and normalized using Athena program, included in the DEMETER package.[8]

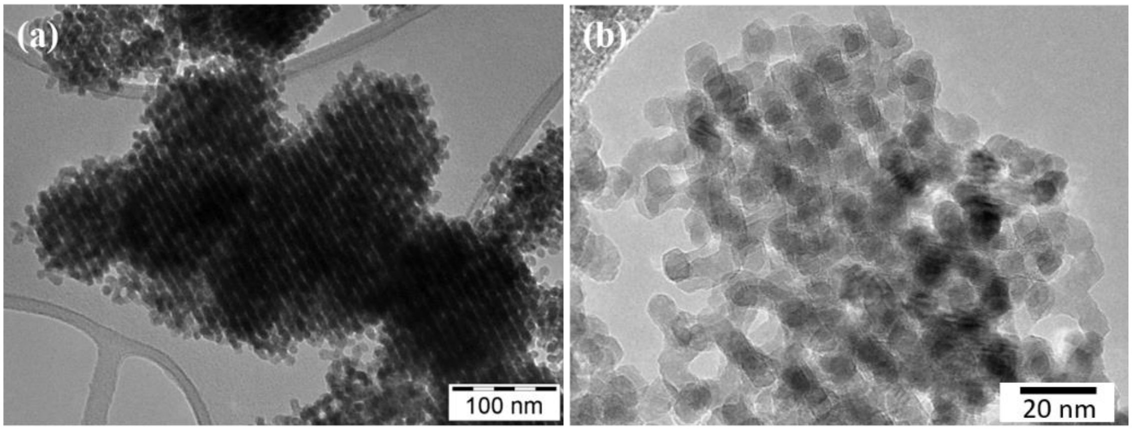

Figure S1. TEM images of nanocast Co_3O_4 using KIT-6 as hard template.

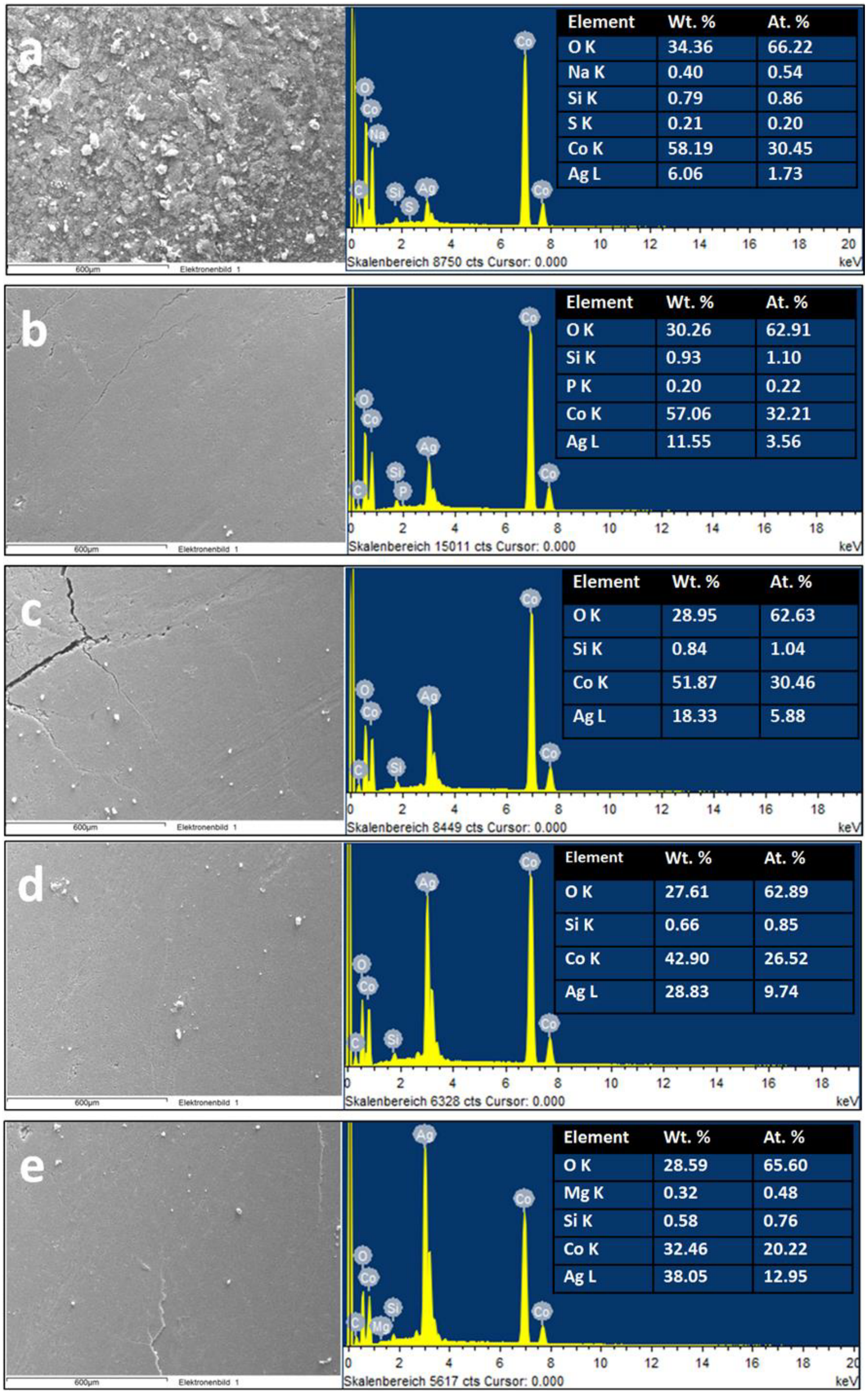

Figure S2. SEM images with corresponding EDX analysis of nanocast Ag-Co oxides: (a) $Co_{16}Ag$ oxide, (b) Co_8Ag oxide, (c) Co_4Ag oxide, (d) Co_2Ag oxide, and (e) CoAg oxide.

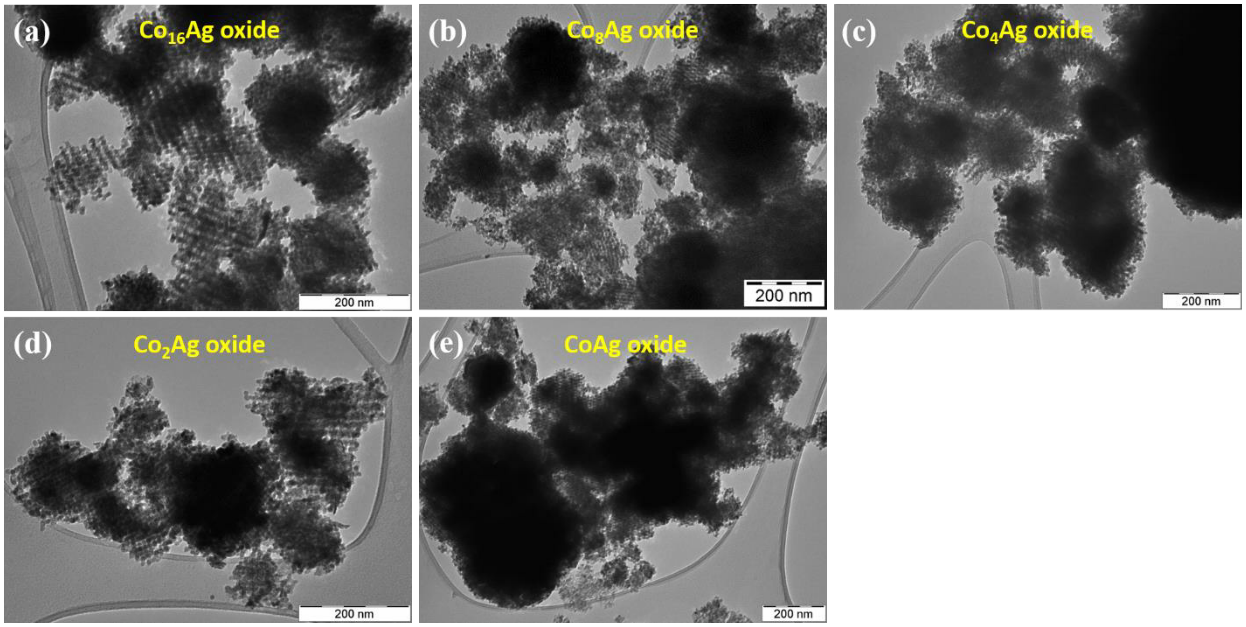

Figure S3. TEM images of nanocast Ag-Co oxides with different Co/Ag ratio: (a) $Co_{16}Ag$ oxide, (b) Co_8Ag oxide, (c) Co_4Ag oxide, (d) Co_2Ag oxide and (e) CoAg oxide.

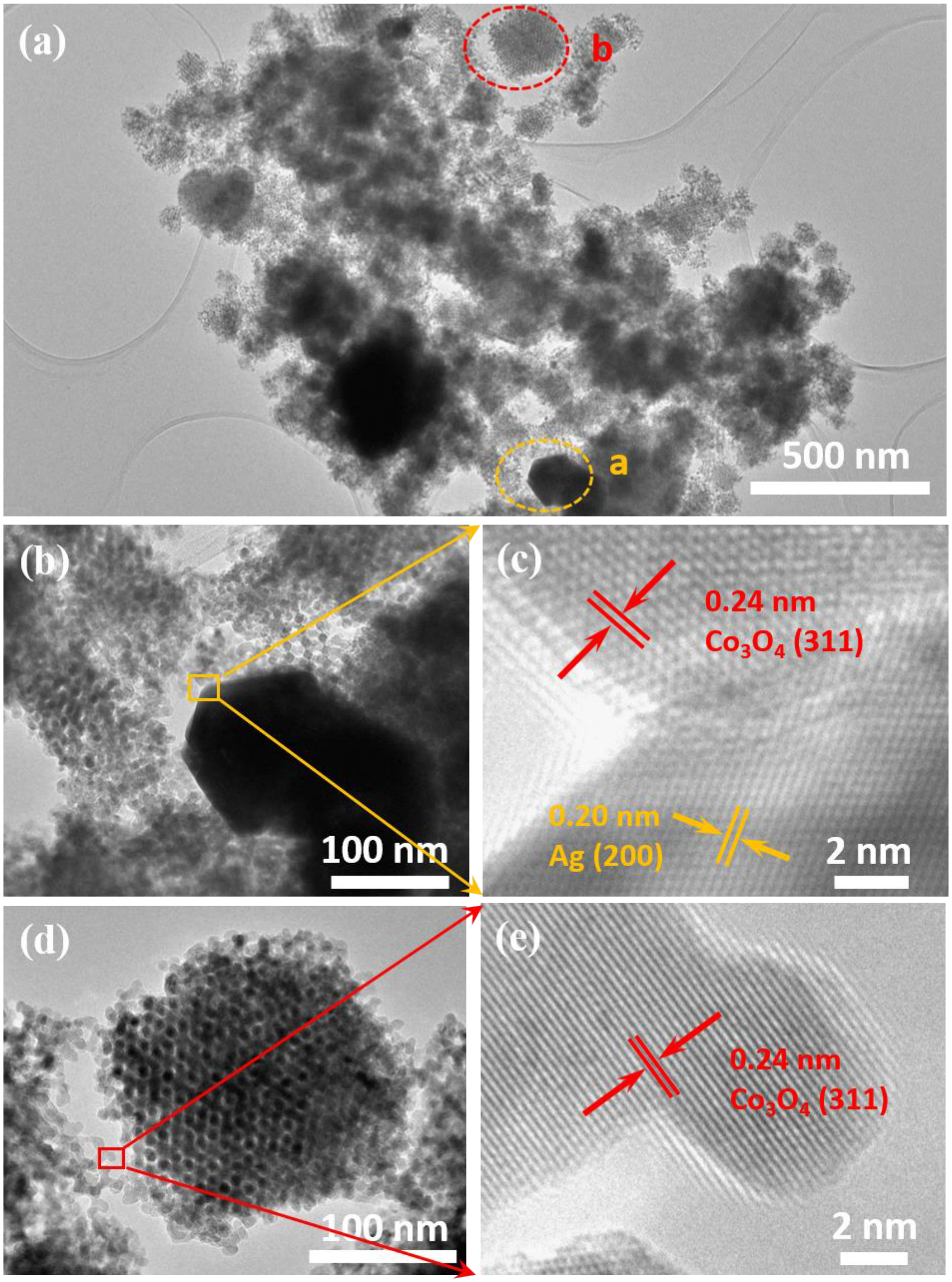

Figure S4. HR-TEM images of a selected oxide (Co/Ag 8:1). (a) an overview image of the oxide. (b) and (d) are the close-up of the marked circle a (orange) and circle b (red) in (a), respectively. (c) and (e) are the close-up of the marked rectangles in (b) and (d), respectively. In Figure S4c, the interface between metallic Ag and spinel Co_3O_4 was shown. The lattice fringes on the large crystal in (b) have a spacing of 0.20 nm, corresponding to the (200) planes of metallic Ag.

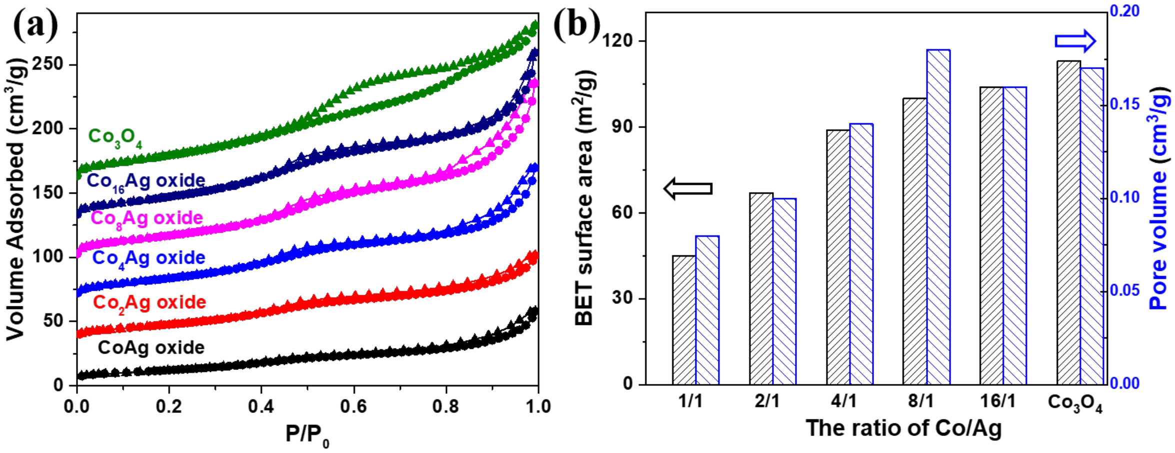

Figure S5. (a) N_2 physisorption isotherms of nanocast Co_3O_4 and Ag-Co oxides, an offset of 30 cm^3/g was applied for a separate display of each isotherm. (b) Summarized value of BET surface area (black columns) and pore volume (blue columns) of nanocast Ag-Co oxides.

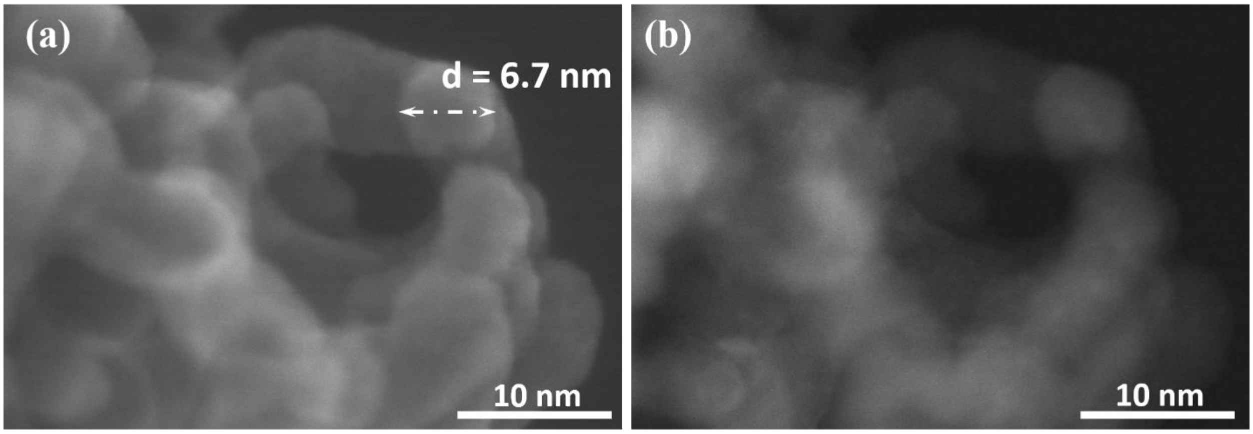

Figure S6. Secondary electron (a) and high-angle annular dark-field STEM image (b) of Co_8Ag oxide. The ordered mesoporous oxide is composed of crystalline particles with diameter of ~ 7 nm.

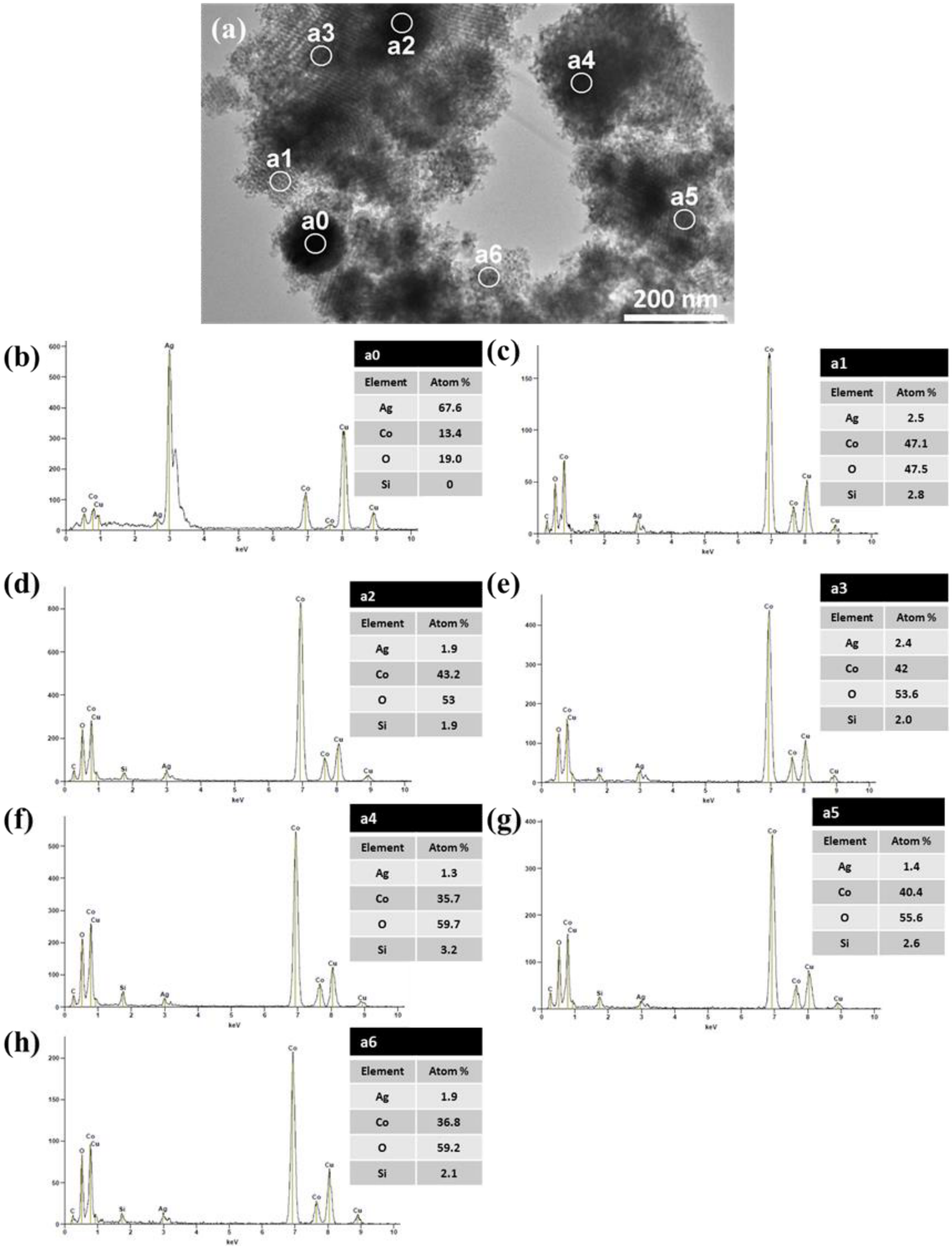

Figure S7. (a) TEM image of Co_4Ag oxide. (b-h) local EDX analysis corresponding to the area marked in (a).

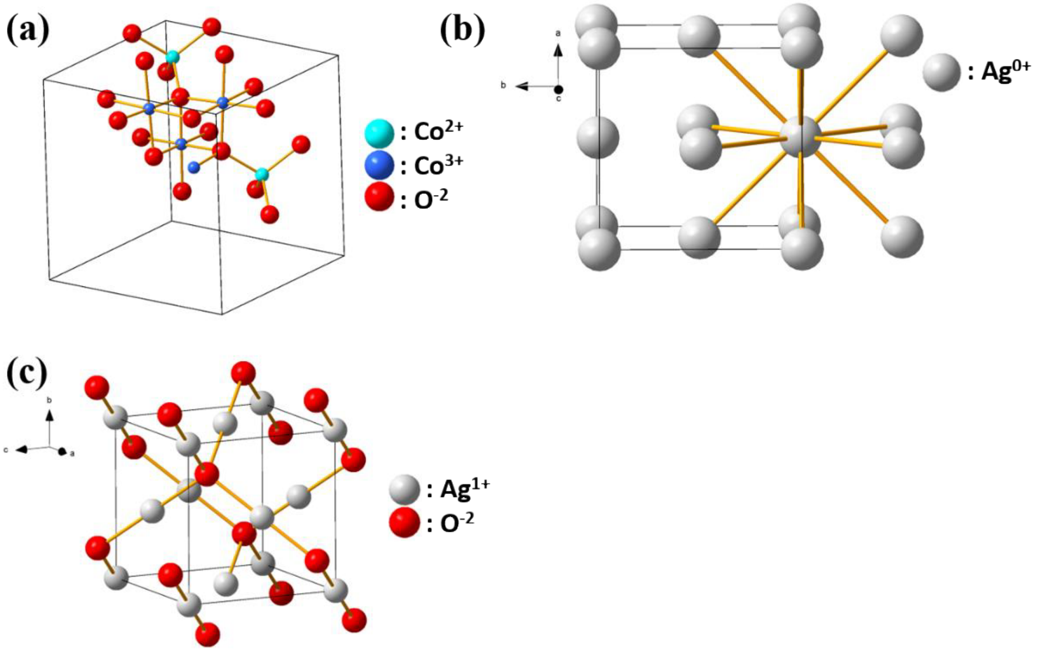

Figure S8. Model crystal structures for: (a) Co_3O_4 spinel, (b) cubic Ag metal, and (c) Ag_2O.

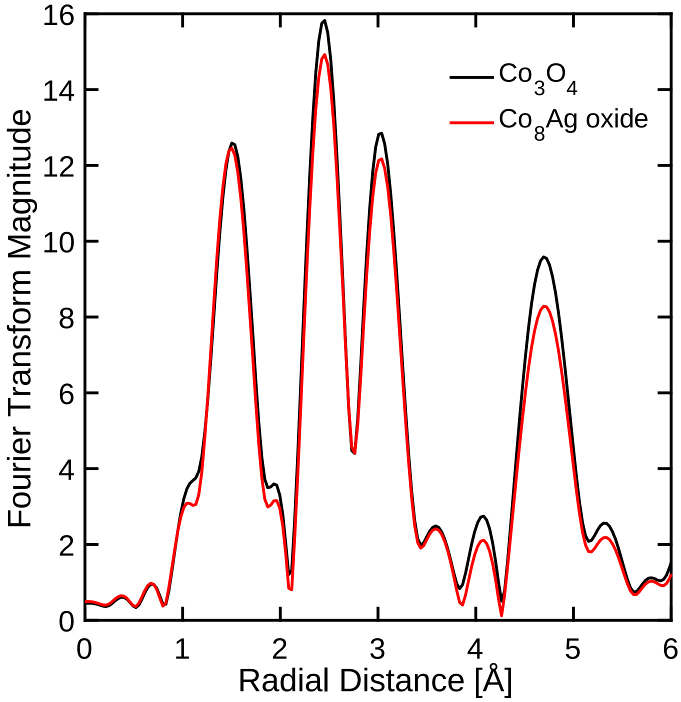

Figure S9. Non-phase-shifted Fourier Transformed of k^3-weighted Co-K edge EXAFS spectra of nanocast Co_3O_4 and Co_8Ag oxide (k-range= 2-13 $Å^{-1}$).

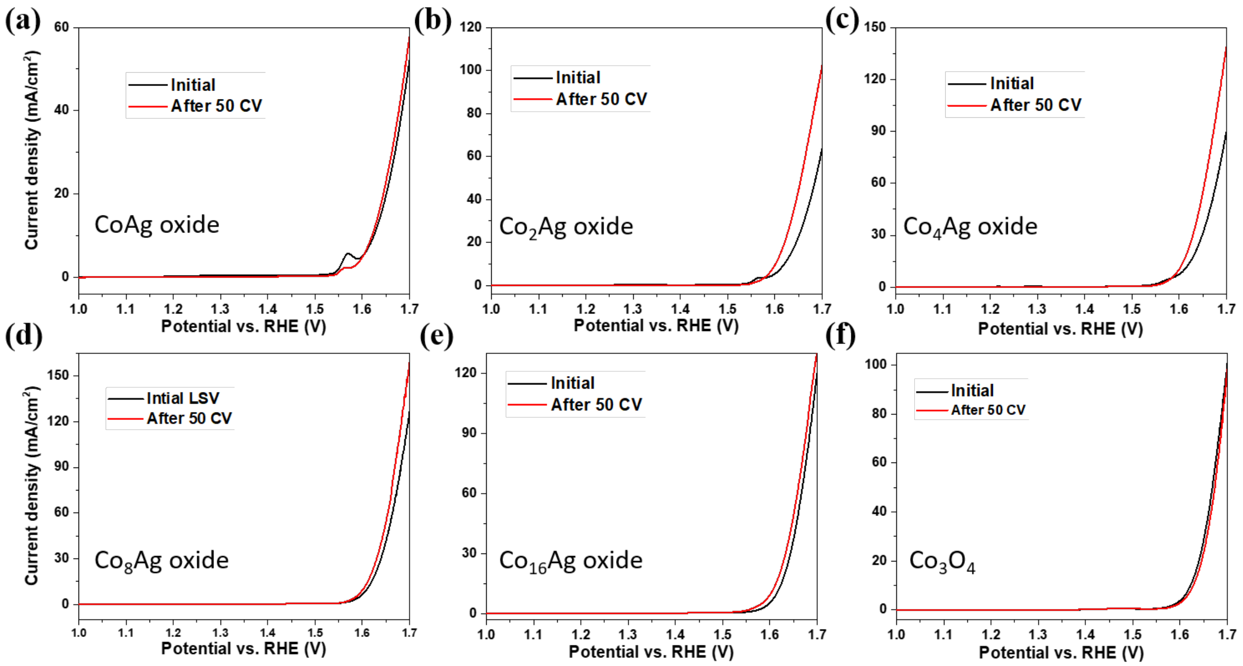

Figure S10. LSV curves of nanocast Ag-Co oxides collected before and after 50 CV: (a) CoAg oxide, (b) Co_2Ag oxide, (c) Co_4Ag oxide, (d) Co_8Ag oxide, (e) $Co_{16}Ag$ oxide, and (e) Co_3O_4. The measurement was conducted in 1 M KOH electrolyte that has Fe impurities.

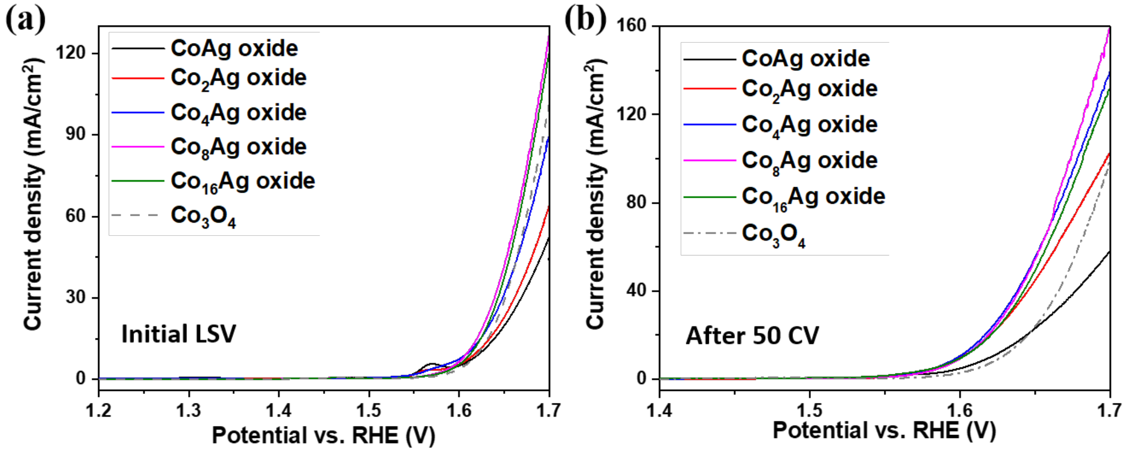

Figure S11. Summarized LSV curves from Figure S5 of nanocast Ag-Co oxides collected before (a) and after (b) 50 CV. The measurement was conducted in unpurified 1 M KOH electrolyte that has Fe impurities.

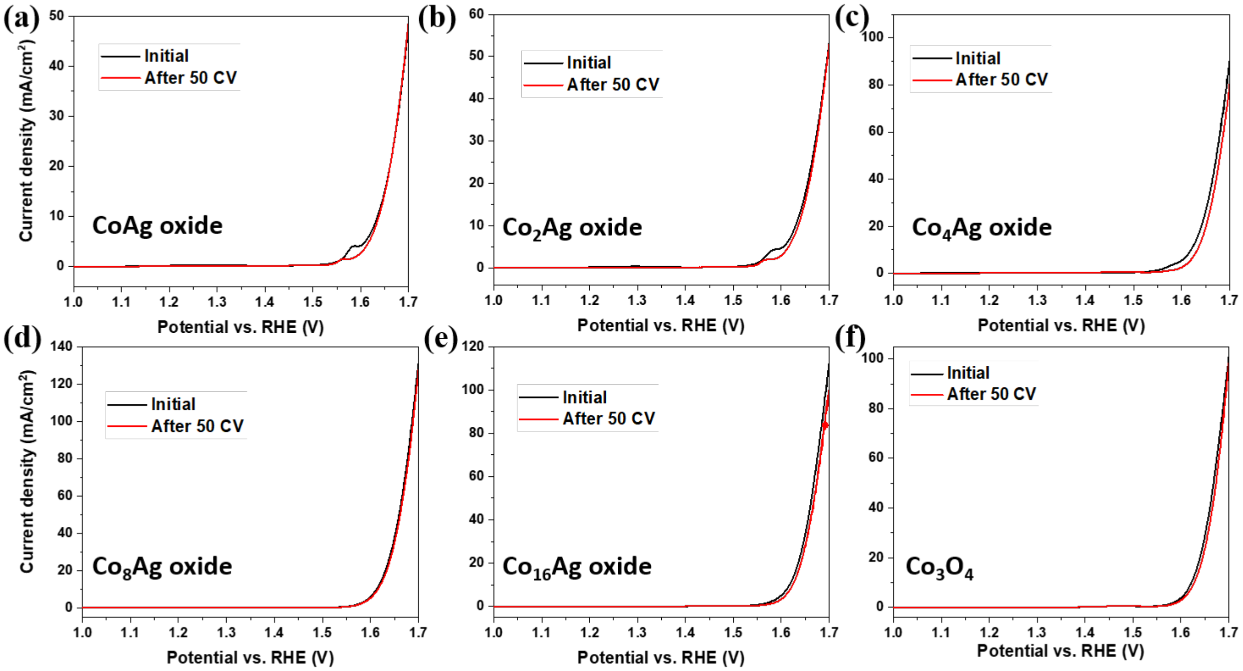

Figure S12. LSV curves of nanocast Ag-Co oxides collected before and after 50 CV: (a) CoAg oxide, (b) Co_2Ag oxide, (c) Co_4Ag oxide, (d) Co_8Ag oxide, (e) $Co_{16}Ag$ oxide, and (e) Co_3O_4. The measurement was conducted in 1 M KOH electrolyte with purification treatment to remove Fe impurities.

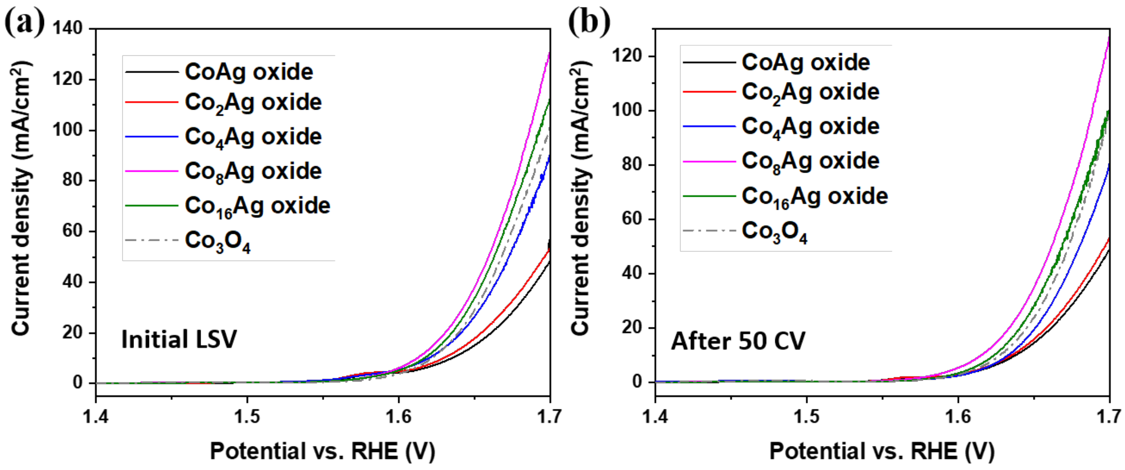

Figure S13. Summarized LSV curves from Figure S7 of nanocast Ag-Co oxides collected before (a) and after (b) 50 CV. The measurement was conducted in 1 M KOH electrolyte with purification treatment to remove Fe impurities.

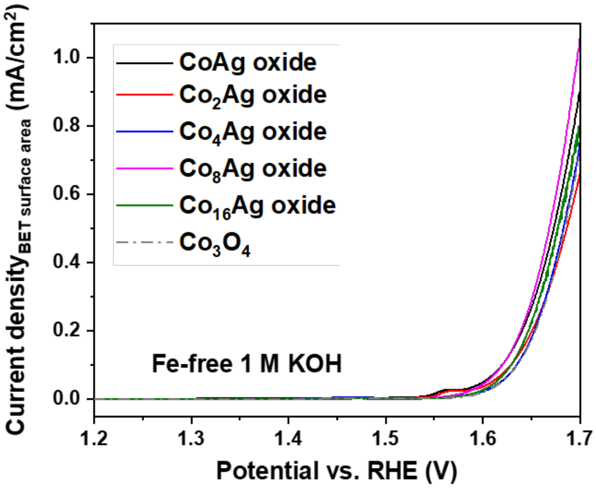

Figure S14. The LSV curves normalized to the BET surface area for nanocast Ag-Co oxide collected before 50 CV. The measurement was conducted in 1 M Fe-free KOH electrolyte.

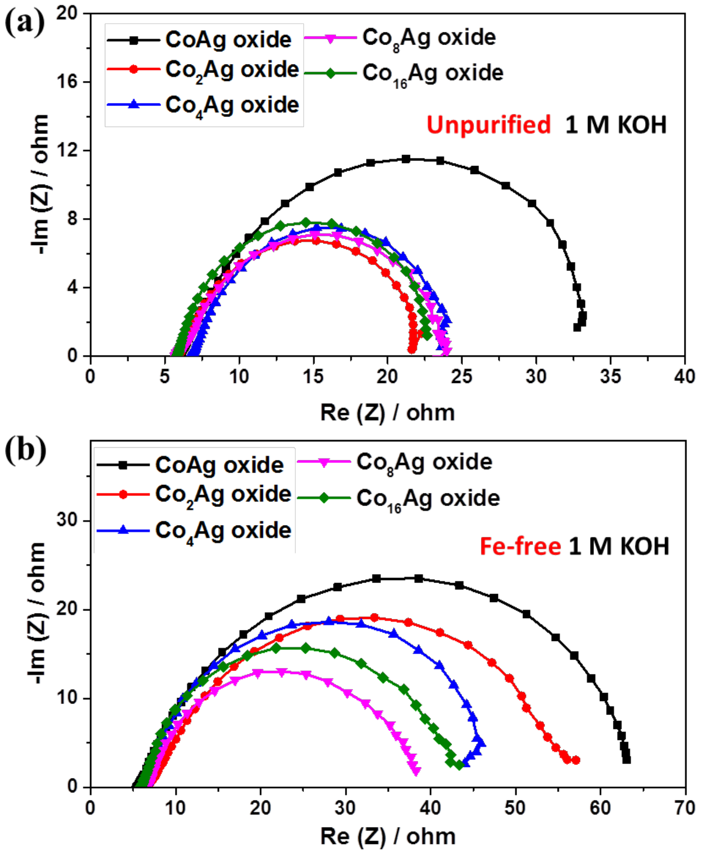

Figure S15. Nyquist plots of nanocast Ag-Co oxides. The EIS measurements were conducted in unpurified (a) and Fe-free 1 M KOH electrolyte (b).

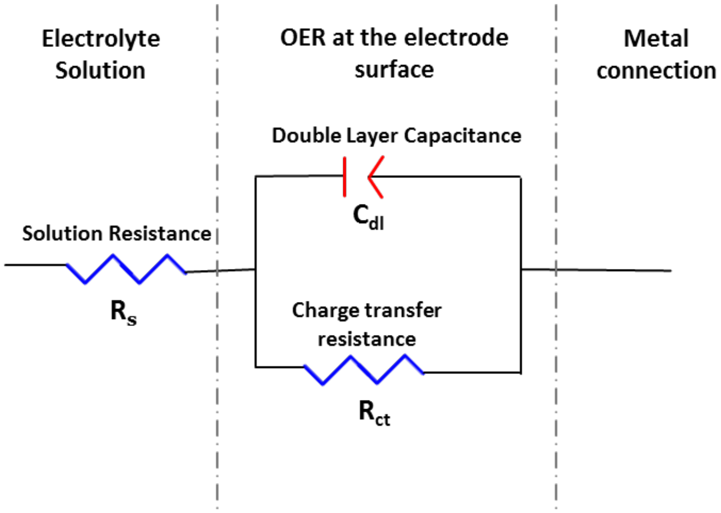

Figure S16. Simplified Randles circuit for the metal oxides catalyzing OER.

In a simplified Randles circuit, R_s and R_{ct} represent the solution resistance and charge transfer resistance, respectively. C_{dl} element models the double-layer capacitance. The kinetics for Faradaic OER is determined by charge transfer resistance (R_{ct}).

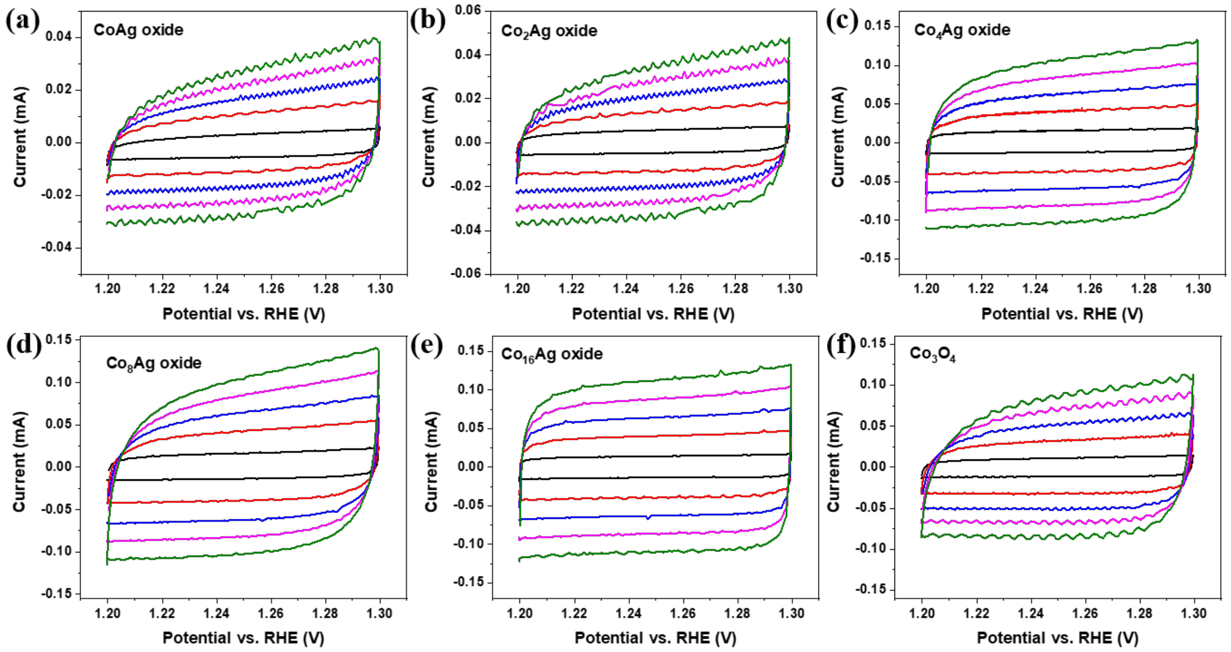

Figure S17. CV curves of curves of nanocast Ag-Co oxides: (a) CoAg oxide, (b) Co_2Ag oxide, (c) Co_4Ag oxide, (d) Co_8Ag oxide, (e) $Co_{16}Ag$ oxide, and (e) Co_3O_4, which were collected in a non-Faradaic region (1.2 – 1.3 V vs RHE) at varying scan rates, i.e., 20 mV/s (black), 60 mV/s (red), 100 mV/s (blue), 140 mV/s (magenta), and 180 mV/s (olive). The measurement was conducted in unpurified 1 M KOH electrolyte after 50 CV scans.

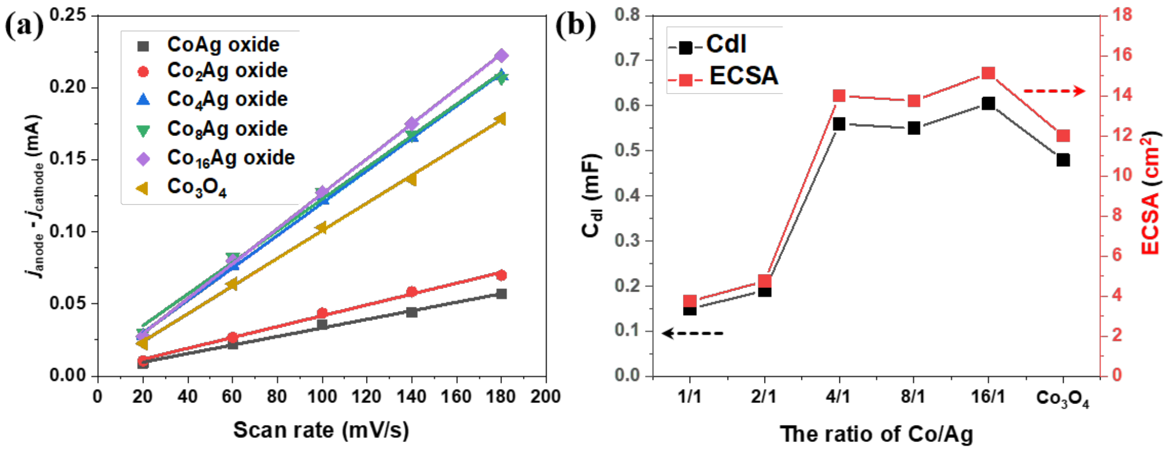

Figure S18. (a) Capacitive current differences (j_{anode} - $j_{cathode}$) at 1.25 V_{RHE} against increasing scan rates and (b) Double-layer capacitance (C_{dl}) and electrochemical surface area (ECSA) of nanocast Ag-Co oxides. The ECSA was calculated according to this equation: ECSA = C_{dl}/Cs, where C_{dl} is specific capacitance, in here 0.04 mF/cm^2 was chosen as the reference value for the measurement in 1 M KOH electrolyte.[9]

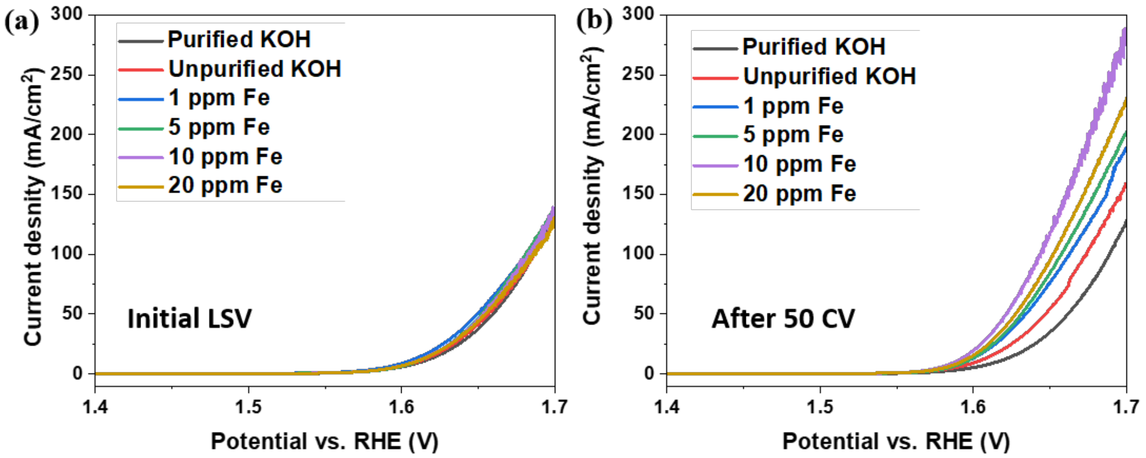

Figure S19. Summarized LSV curves of Co_8Ag oxide collected before (a) and after (b) 50 CV. The measurement was conducted in 1 M KOH electrolyte with varying concentration of Fe impurity.

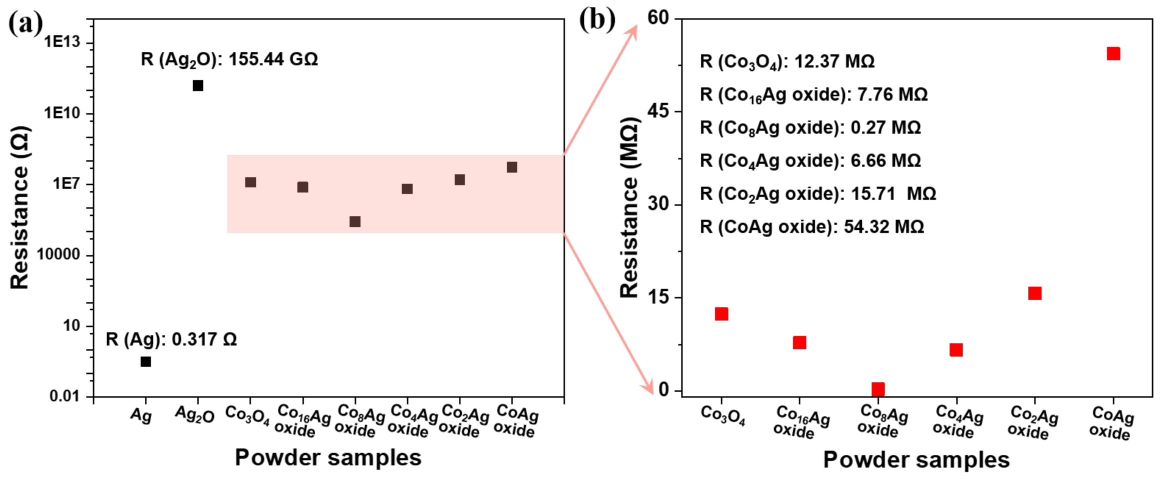

Figure S20. Electrical resistance comparison of of Co_3O_4, Co_xAg oxide (x = 16, 8, 4, 2, and 1), silver nanoparticles, and silver oxide (Ag_2O) powders using a homemade cell.

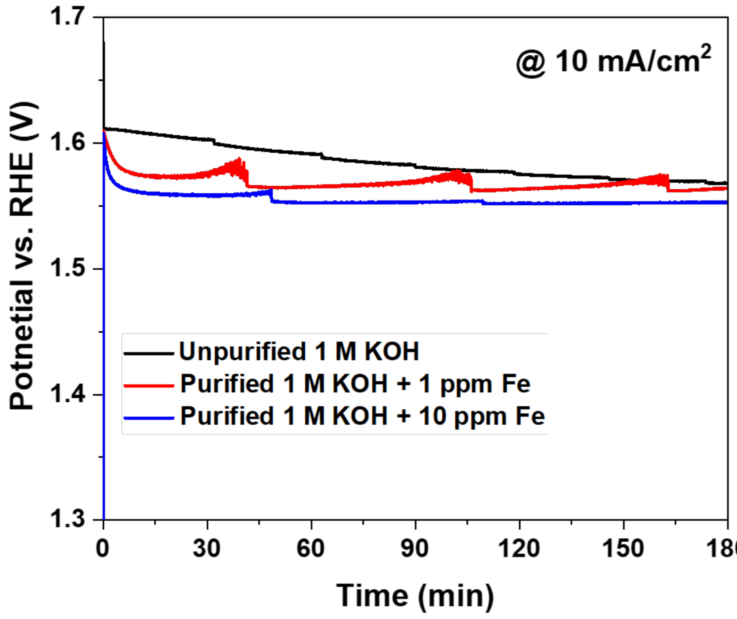

Figure S21. Chronopotentiometric curves at a current density of 10 mA/cm^2 of Co_8Ag oxide in unpurified1 M KOH and purified KOH electrolyte with adding 1 and 10 ppm Fe.

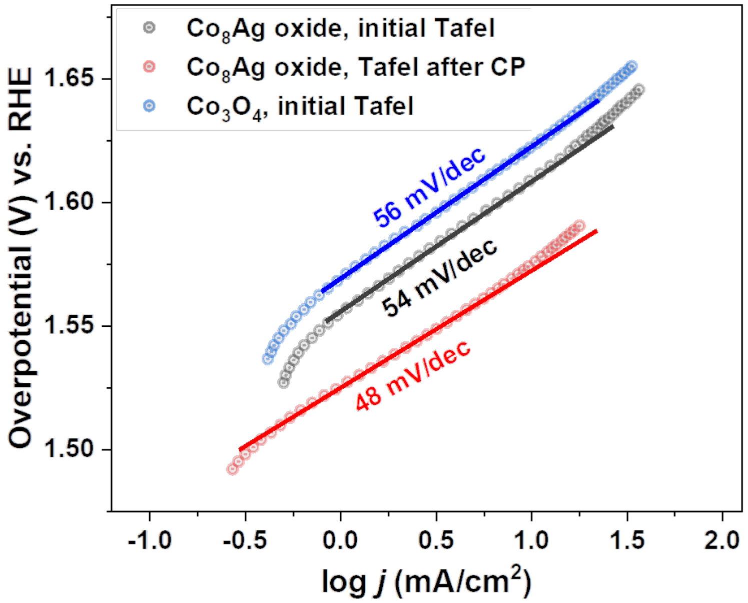

Figure S22. Tafel slopes of initial Co_8Ag oxide, activated Co_8Ag oxide after CP, and initial Co_3O_4.

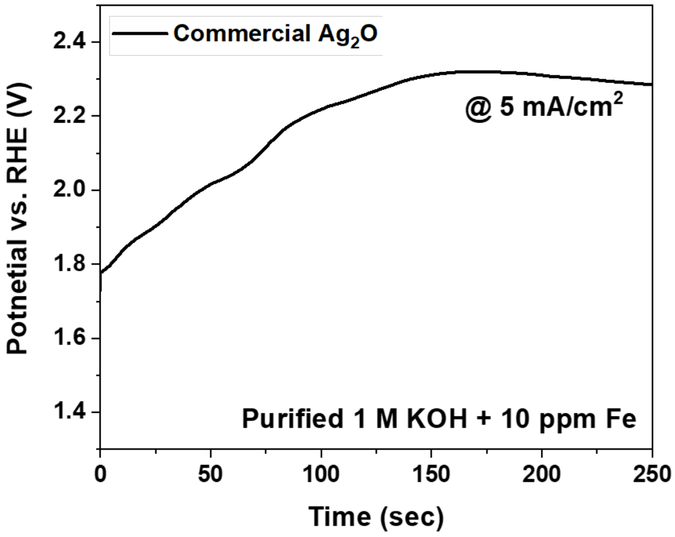

Figure S23. Chronopotentiometry curve of Ag_2O collected at a current density of 5 mA/cm^2 in 1M KOH electrolyte with 10 ppm Fe. Due to the insulating behavior of Ag_2O, the high potential was required to reach at 5 mA/cm^2.

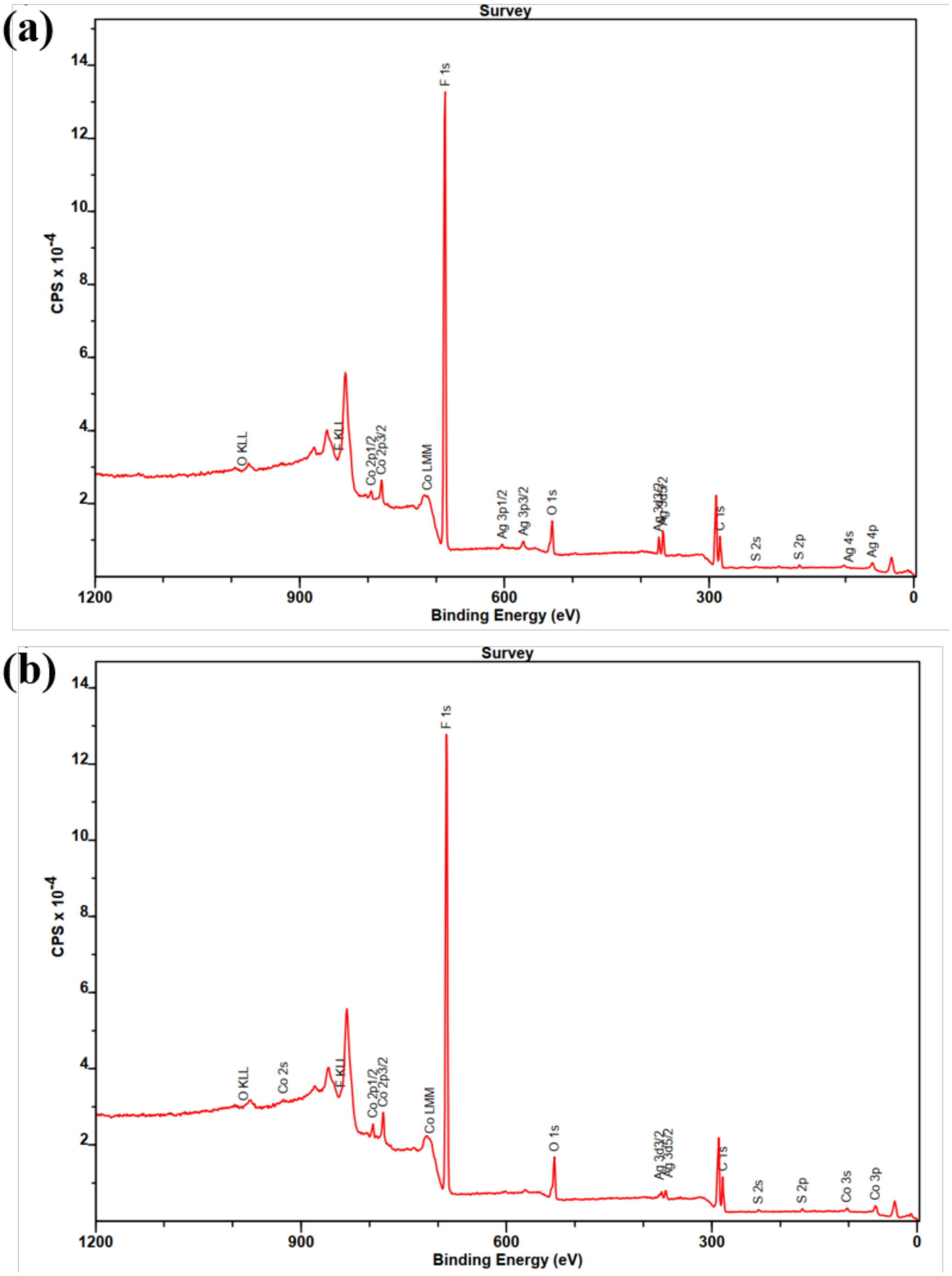

Figure S24. XPS survey of Co_8Ag oxide@carbon fiber paper before and after OER stability test. C, S, and F were detected on the electrode, which were from Nafion.

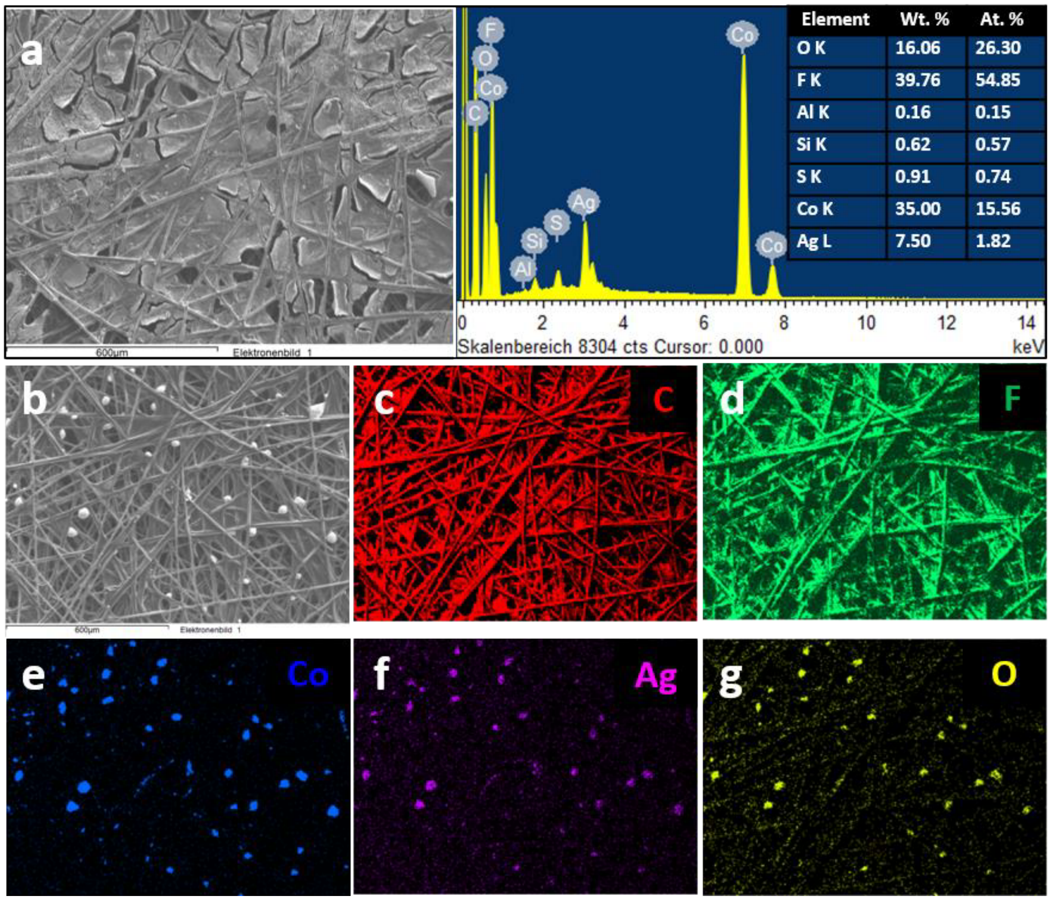

Figure S25. (a) SEM image with corresponding EDX analysis of Co_8Ag oxide deposited on carbon fiber paper using Nafion binder, (b) SEM image and corresponding elemental mapping images of (c) carbon, (d) fluorine, (e) cobalt, (f) silver, and (g) oxygen. Note: EDX analysis excluded the content of carbon. S and F elements were due to Nafion, and a small amount of Al came from the sample holder.

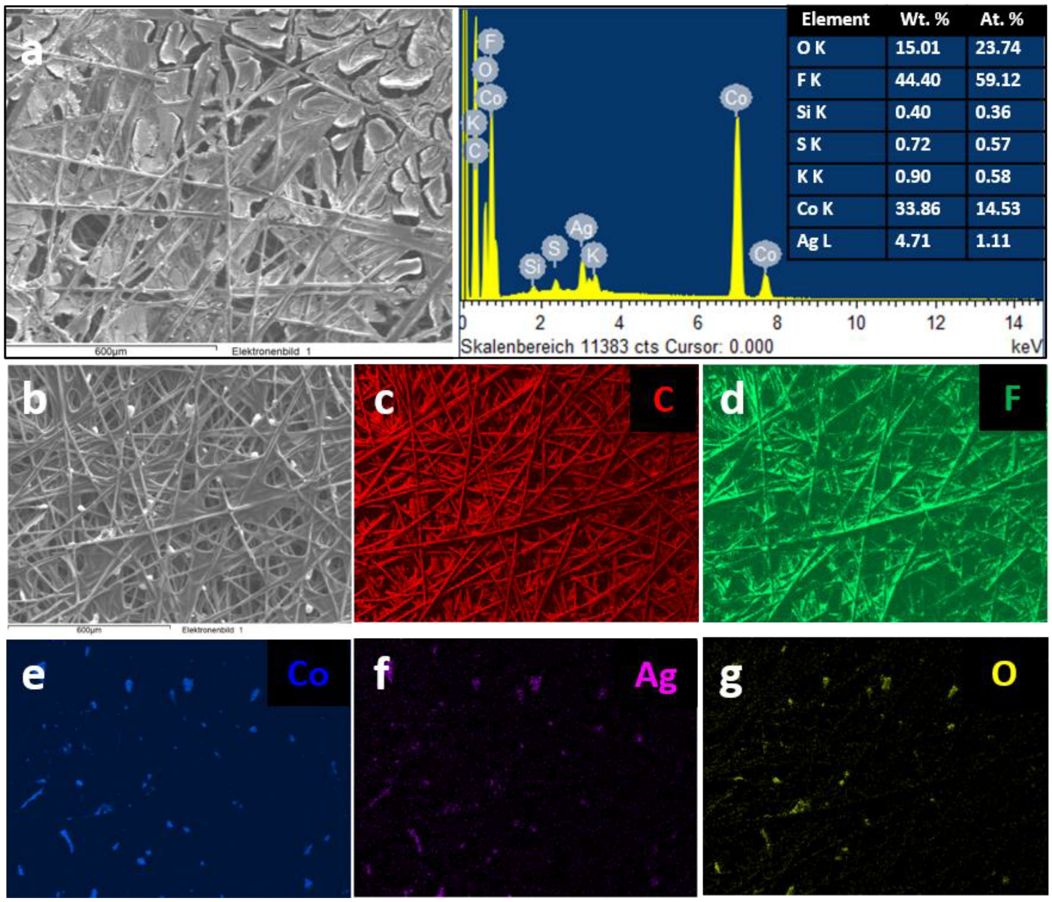

Figure S26. (a) SEM image with corresponding EDX analysis of Co_8Ag oxide deposited on carbon fiber paper which was collected after the chronopotentiometry for 12 h, (b) SEM image and corresponding elemental mapping images of (c) carbon, (d) fluorine, (e) cobalt, (f) silver, and (g) oxygen. Note: EDX analysis excluded the content of carbon. A small amount of K was due to the residue of KOH on the electrode.

Table S1. The Co/Ag ratio and textural parameters of nenocasted Co-Ag oxides, derived from EDX and nitrogen phsisorption, respectively.

	Co_3O_4	$Co_{16}Ag$ oxide	Co_8Ag oxide	Co_4Ag oxide	Co_2Ag oxide	CoAg oxide
Actual ratio of Co/Ag	-	17.6 : 1	9.0 : 1	5.2 : 1	2.7 : 1	1.6 : 1
BET surface area (m^2/g)	113	104	100	89	67	45
Pore volume (cm^3/g)	0.17	0.16	0.19	0.14	0.10	0.08

Table S2. OER performanc of cobalt based electrocatalysts reported in recent literatures.

Catalyst	Electrolyte	Loading (mg/cm^2)	Tafel slope (mV/dec)	η at *j* = 10 mA/cm^2 (mV)	Reference
Co_3O_4/N-rmGO@Ni foam	0.1 M KOH	1	42	310	[10]
$Co(NO_3)_2$ $6H_2O$	1 M KOH	0.12	55.9	323	[11]
$CoFe_2O_4$/CoO	1 M KOH	0.2	71	320	[12]
Co_3O_4/$NiCo_2O_4$@Ni foam	1 M KOH	1	88	340	[13]
Activated Co_8Ag oxide	1 M KOH	0.12	48	344	This work
Co_3O_4 NPs/graphene	1 M KOH	0.19	56	350	[14]
$Co_{2.25}Cr_{0.75}O_4$	1 M NaOH	0.84	60 ± 3	350 ± 10	[15]
$CoFe_2O_4$ nanorods	1 M KOH	0.19	96	355	[16]
STL templated Co_3O_4	1 M KOH	0.12	45-53	401	[17]
Reduced Co_3O_4 nanowires	1 M KOH	0.136	72	400	[18]
Nanoparticulate Co_3O_4	1 M NaOH	0.8	60.9	500 ± 10	[19]

Reference:

[1] X. H. Deng, K. Chen, H. Tuysuz, *Chem. Mater.* **2017**, *29*, 40-52.

[2] L. Trotochaud, S. L. Young, J. K. Ranney, S. W. Boettcher, *J. Am. Chem. Soc.* **2014**, *136*, 6744-6453.

[3] P. Juhas, T. Davis, C. L. Farrow, S. J. L. Billinge, *J. Appl. Crystallogr.* **2013**, *46*, 560-566.

[4] C. L. Farrow, P. Juhas, J. W. Liu, D. Bryndin, E. S. Božin, J. Bloch, T. Proffen, S. J. L. Billinge, *J. Phys.: Condens. Matter* **2007**, *19*, 335219.

[5] J. Spreadborough, J. W. Christian, *J. Sci. Instrum.* **1959**, *36*, 116-118.

[6] S. Srivastava, R. Srivastava, S. Pandey, *J. Phys. Soc. Jpn.* **1977**, *43*, 1463-1464.

[7] P. Meena, R. Kumar, K. Sreenivas, *AIP Conf. Proc.* **2013**, *1512*, 1204.

[8] B. Ravel, M. Newville, *J. Synchrotron Radiat.* **2005**, *12*, 537-541.

[9] C. C. McCrory, S. Jung, I. M. Ferrer, S. M. Chatman, J. C. Peters, T. F. Jaramillo, *J. Am. Chem. Soc.* **2015**, *137*, 4347-4357.

[10] Y. Liang, Y. Li, H. Wang, J. Zhou, J. Wang, T. Regier, H. Dai, *Nat. Mater.* **2011**, *10*, 780-786.

[11] G. H. Moon, M. Yu, C. K. Chan, H. Tuysuz, *Angew. Chem. Int. Ed. Engl.* **2019**, *58*, 3491-3495.

[12] F. Waag, B. Gokce, C. Kalapu, G. Bendt, S. Salamon, J. Landers, U. Hagemann, M. Heidelmann, S. Schulz, H. Wende, N. Hartmann, M. Behrens, S. Barcikowski, *Sci. Rep.* **2017**, *7*, 13161.

[13] H. Hu, B. Guan, B. Xia, X. W. Lou, *J. Am. Chem. Soc.* **2015**, *137*, 5590-5595.

[14] Y. Zhao, S. Chen, B. Sun, D. Su, X. Huang, H. Liu, Y. Yan, K. Sun, G. Wang, *Sci. Rep.* **2015**, *5*, 7629.

[15] C.-C. Lin, C. C. L. McCrory, *ACS Catal.* **2016**, *7*, 443-451.

[16] Y. Ding, J. Zhao, W. Zhang, J. Zhang, X. Chen, F. Yang, X. Zhang, *ACS Appl. Energy Mater.* **2019**, *2*, 1026-1032.

[17] X. Deng, C. K. Chan, H. Tuysuz, *ACS Appl. Mater. Interfaces* **2016**, *8*, 32488-32495.

[18] Y. C. Wang, T. Zhou, K. Jiang, P. M. Da, Z. Peng, J. Tang, B. A. Kong, W. B. Cai, Z. Q. Yang, G. F. Zheng, *Adv. Energy Mater.* **2014**, *4*, 1400696.

[19] F. Song, X. Hu, *J. Am. Chem. Soc.* **2014**, *136*, 16481-16484.
